# Multiple directed mutagenesis reduces enzymatic activity and antibody recognition of the African Swine Fever Virus E2 ubiquitin-conjugating protein (ASFV-pI215L)

**DOI:** 10.1080/22221751.2026.2622218

**Published:** 2026-01-23

**Authors:** Nuno Jordão, Ana Catarina Urbano, Fernando Boinas, Carlos Martins, Fernando Ferreira

**Affiliations:** a CIISA – Centre for Interdisciplinary Research in Animal Health, Faculty of Veterinary Medicine, University of Lisbon, Lisbon, Portugal; b Laboratory for Animal and Veterinary Sciences (AL4AnimalS), Lisbon, Portugal

**Keywords:** ASFV, pI215L, mutagenesis, vaccine, DIVA

## Abstract

African Swine Fever virus (ASFV) causes a contagious and fatal disease in domestic pigs and Eurasian wild boars, representing a serious threat to the global pig industry, since no antivirals are available and vaccine use is currently restricted to Vietnam. Notably, ASFV encodes for an E2 ubiquitin-conjugating enzyme (ASFV-pI215L) which is essential for viral replication and evasion from immune interferon type I responses, suggesting that its functional impairment could lead to a live attenuated vaccine. In this study, we showed that ASFV-pI215L is highly conserved among 222 ASFV isolates, including the emerging ones, emphasizing its value as a target for vaccine design. Furthermore, our mutagenic studies revealed that single- and multiple-residue substitutions comprising the R11-E15 and D130-S134 residues reduced ASFV-pI215L E2 ubiquitin-conjugating activity. In parallel, a strong immunodominant B-cell epitope was mapped and mutated between P61 and F69 resides, reducing or abolishing both IgG and IgM recognition, and ASFV-pI215L E2 activity. In sum, this study highlights that rational targeted mutagenesis can reduce E2 ubiquitin-conjugating activity and immune recognition of ASFV-pI215L, providing a strategy to develop an attenuated vaccine able to differentiate infected from vaccinated animals.

## GRAPHICAL ABSTRACT

**Figure:**
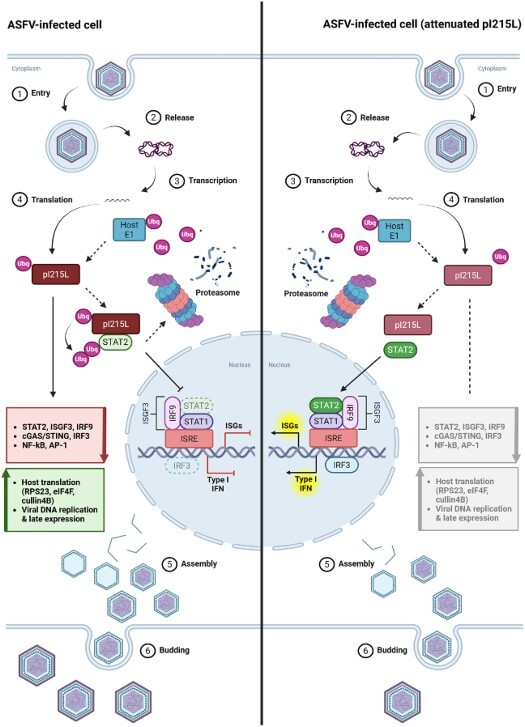

## Introduction

African swine fever (ASF) is a viral infectious disease that affects domestic pigsand Eurasian wild boars, showing a mortality rate close to 100% in acute forms. ASF was present in Western Europe from the late 50s to the early 90s, when it was successfully eradicated except in Sardinia. In 2007, ASF reemerged in the Caucasus region, having reached the European Union in 2014 and China in 2018, then spreading across Southeast Asia, Oceania and America [1,2]. Since no antiviral treatment is available and existing vaccines, based on viral attenuation, are restricted for use in Vietnam due to safety and efficiency concerns, ASF control currently still relies on strict sanitary measures [2–5].

African swine fever virus (ASFV) targets the monocyte – macrophage cell lineage, displaying a 9-hour infectious cycle divided into an early stage (preceding viral DNA replication in the cytoplasm) and a late stage (featuring a buildup of viral products at perinuclear factories). ASFV carries a dsDNA genome of 170–200 kbp that contains between 150 and 167 open reading frames (ORF), depending of the virus strain [2,5,6–8]. The ORF I215L, located at 15 kbp from the right terminus, encodes the protein ASFV-pI215L, with a sequence and function akin to ubiquitin-conjugating (E2) enzymes [9–11].

E2 enzymes are part of the ubiquitin-proteasome system (UPS), a eukaryotic signalling mechanism in which ubiquitin chains are bound to proteins, mostly for proteasome degradation. Initially, ubiquitin-activating (E1) enzymes form a thioester bond with ubiquitin and transfer it to E2 enzymes, after which ubiquitin is transferred from E2 to a protein target presented by ubiquitin ligase (E3) enzymes. After multiple cycles, targets carry polyubiquitin chains built with lysine linkage and structure according to the purpose of ubiquitination. Several viruses express proteins that are able to mimic or interact with UPS components, to avoid host immune response and improve viral production [12,13].

Under *in vitro* conditions, ASFV-pI215L can receive ubiquitin from swine and human E1, and ubiquitinates histones and other E2 enzymes in the absence of E3 [9,14]. ASFV-pI215L forms a thioester link to ubiquitin through one active site, cysteine 85 (C85), transports mono or di-ubiquitin, and mediates the formation of polyubiquitin chains with K48 linkage (for proteasomal degradation) or K63 linkage (associated with NF-kB signalling, DNA repair and lysosomal degradation) [13–15]. Besides its ubiquitin-conjugating core (UBC) domain, ASFV-pI215L possesses an acidic C-terminal tail extension that may be relevant for target recognition and ubiquitination without E3 enzymes [10,16].

Transcription of the ORF I215L starts at 2 h post-infection (hpi), with gene knockdown experiments resulting in a decrease of viral DNA replication, late protein expression and progeny. ASFV-pI215L expression is also detectable from 2 hpi and is considered an early protein, showing a nuclear pattern which suggests modulation of DNA repair or antiviral response. In parallel, ASFV infection induces degradation of ubiquitin mRNA before and during viral DNA replication [14,15,17,18]. At the late stage, ASFV-pI215L expression increases and migrates to viral factories, where is also detected K48 and K63-polyubiquitin, usually found mostly in the nucleus. Moreover, proteasome activity is promoted and diverted to viral factories for enhancement of viral replication, late translation and egress [14,19,20]. Although ASFV-pI215L presence in the extracellular virion is debated, several ubiquitinated viral proteins have been detected, including the core shell p15, which results from the possibly ubiquitin-related cleavage of pp62 [6,19,21].

The targets of ASFV-pI215L ubiquitination remain largely unknown but many interactions independent of E2 activity have been found, such as the nuclear protein SMCp which contains a domain that interacts with A/T rich DNA regions [17]. ASFV-pI215L could also mediate cellular translation by interacting with the 40S ribosomal subunit protein RPS23, the translation initiation factor eIF4E and the mTOR signalling-associated E3 enzyme Cullin 4B, all found to be upregulated during ASFV infection and colocalized with ASFV-pI215L in the nucleus and viral factories [15].

In parallel, ASFV-pI215L helps evading the host innate antiviral response through inhibition of type I interferon (IFN-I) responses, by inducing lysosomal degradation of cellular IRF9, which hampers ISGF3 formation, its migration to the nucleus and transcription of IFN-stimulated genes (ISGs) responsible for antiviral responses [22,23]. ASFV-pI215L also colocalizes and ubiquitinates STAT2 for proteasomal degradation, further inhibiting ISGs IFIT1 and ISG15 [24]. Moreover, ASFV-pI215L is the strongest contributor to viral suppression of the (cGAS)/STING signalling pathway, promoter of IFN-I response, through its interaction with the E3 enzyme RNF138, as demonstrated by the increased IFN-β levels after ASFV-pI215L knockdown [25]. ASFV-pI215L is also linked to the inhibition of antiviral transcription factors NF-κB and AP-1, with NF-κB being further inhibited by the ASFV-pI215L interaction with IKKβ and blockage of p65 nuclear migration after cytokine stimulation [26]. Notably, ASFV-pI215L also induces secretion of type II interferon IFN-γ by T-cell lymphocytes of ASFV-immune pigs, and some animals present B-cell production of IgG that recognizes ASFV-pI215L [27].

Thus, considering the reported functions of ASFV-pI215L during viral infection cycle and on the regulation of host immune responses, a bioinformatic study was conducted to better understand the evolutionary origin of ASFV-pI215L and its conservation amongst ASFV isolates of different genotypes. Then, the modulation of ASFV-pI215L enzymatic activity and immunogenicity was explored through mutagenic studies, revealing that multiple-residue missense mutations disrupt ASFV-pI215L E2 activity and B-cell epitope recognition. Altogether, our results strongly suggest that rational site-directed mutagenesis may generate efficient live attenuated ASFV vaccine candidates with DIVA (differentiating infected from vaccinated animals) capabilities.

## Materials and methods

### Bioinformatic analysis of ASFV-pI215L

For comparison of ASFV-pI215L with other E2 enzymes, the ASFV-pI215L sequence from the Ba71 V strain (Uniprot accession number P27949) was employed as the query sequence for multiple BLASTP analysis (version 2.16.0), restricted to selected organisms, resulting in the collection of 24 eukaryotic and 10 viral ubiquitin-conjugating proteins (Supplementary Table S1). An additional set of 22 ubiquitin-conjugating proteins encoded by *Asfarviridae* or *Poxviridae* was retrieved from an NCBI Protein word search and TBLASTN homology search (Supplementary Table S2). For intraspecies analysis, 222 ASFV genomes were identified in the NCBI Nucleotide database and ASFV-pI215L sequences were retrieved from genome annotation or homology inferred using a TBLASTN search (Supplementary Table S3). Protein sequences were uploaded to MEGA X, version 10.2.4, for MUSCLE alignment and construction of phylogenetic trees. The evolutionary history was inferred using the Maximum Likelihood statistical method with the JTT substitution model [28], and node support was assessed by executing 1000 bootstrap replications. Protein alignments were obtained with Uniprot Align, information regarding the active site and UBC domain was obtained from the Uniprot protein database (accession number P27949, P49427, P60604, I3LJ21) or homology inferred. Linear B-cell epitopes were predicted with the SVMTriP online tool [29], querying the Ba71 V ASFV-pI215L protein with epitope lengths of 10 or 20 residues. The crystal structure of genotype II ASFV-pI215L was retrieved from RCSB Protein Data Bank (PDB ID 7WLH) [30], where we also obtained information regarding ASFV-pI215L’s secondary structure, with 3D visualization performed using PyMOL, version 3.0.4 (Schrödinger).

### Production of recombinant ASFV-pI215L

After designing the sequences for the ASFV-pI215L mutants (Supplementary Table S4), wildtype and mutant I215L DNA sequences were synthesized and inserted into pET-24a + plasmids (Azenta, Netherlands) for expression of histidine-tagged protein. Each plasmid was transformed into competent *E. coli* BL21 (DE3)-pLysS (Novagen), recombinant bacteria were grown overnight on LB agar (NZYtech) with 30 µg/mL of kanamycin (Sigma-Aldrich) and 34 µg/mL of chloramphenicol (Sigma-Aldrich), followed by overnight growth in LB Broth (Sigma-Aldrich). When each culture reached an 600 nm optical density of 0.4, 1 mM of isopropyl-β-D-thiogalactopyranoside (Sigma-Aldrich) was added to induce ASFV-pI215L expression for 5 h. The bacterial pellet was resuspended in ligation buffer [50 mM Na2HPO4 (Carlo Erba Reagents) and 300 mM NaCl (Sigma-Aldrich), pH 7.4] supplemented with 0.2 mg/mL lysozyme (Sigma-Aldrich), 20 μg/mL DNase I (Roche), and cOmplete® EDTA-free protease inhibitor cocktail (Roche). Each lysate was sonicated (15 min, 70% amplitude, 50% on–off cycles), centrifuged and 0.45 µm syringe filtered. Protein extract was loaded onto His GraviTrap TALON® columns (Cytiva), washed two times with ligation buffer containing 5 mM imidazole (Sigma-Aldrich), and recombinant ASFV-pI215L was eluted using ligation buffer with 150 mM imidazole. Collected protein was placed onto Amicon® Ultra Centrifugal Filters of 10 kDa cut-off (Merck) for concentration and buffer exchange to pH 7.6 PBS (Santa Cruz Biotechnology) with 5% glycerol (Sigma-Aldrich), for – 80°C storage. Produced proteins were quantified using Pierce™ 660 nm Protein Assay Reagent (Thermo Scientific).

### ASFV-pI215L ubiquitination assay

Analysis of functional impairment of produced ASFV-pI215L was adapted from previous work [14], using an E2-Ubiquitin Conjugation Kit (Abcam, ref. ab139472). Briefly, 2 µL of ubiquitination buffer, 2 µL of biotinylated ubiquitin solution, 1 µL of E1 solution and 1 µL of Mg-ATP solution provided by the kit were mixed with 2 µL of inorganic pyrophosphatase (Roche) and 0.05 nmol of recombinant ASFV-pI215L. Each mix was incubated at 37°C for 2 min or 1 h, then added 20 µL of 2× non-reducing gel loading buffer (Abcam). Protein samples were separated in a 12.5% acrylamide gel using a Mini-PROTEAN® Tetra Cell electrophoresis system (Bio-Rad), followed by protein transference to a 0.45 µm PVDF membrane (Amersham). After overnight incubation at 4°C in Blocking Buffer [PBS with 0.1% (v/v) of Tween-20 (Merck) and 5% (w/v) of Bovine Serum Albumin (BSA) (Serana Europe)] and three washes of 5 min each with Washing Buffer [PBS with 0.1% (v/v) of Tween-20], membranes were incubated at room temperature for 1 h with a 1/5000 dilution of Amersham™ Streptavidin-HRP Conjugate (Cytiva, ref. RPN1231) in Blocking Buffer. After five washes, membranes were incubated for 5 min with Clarity Western ECL Substrate (Bio-Rad). Band detection and peak intensity quantification was performed in a ChemiDoc Gel Imaging System (Bio-Rad), and graphs were generated in GraphPad Prism, version 9.0 (GraphPad software).

### ELISA

For B-cell epitope mapping, two peptide libraries were synthesized (Pepscan) containing consecutive and overlapping segments of ASFV-pI215L: a set of 25-residue peptides tagged with biotin, and a second set of PS-tagged 9-residue peptides (Supplementary Tables S5 and S6). All peptides were solubilized *ad hoc* in water, PBS or DMF. Initially, 10 µg of recombinant ASFV-pI215L or 100 nmol of PS-tag peptides were diluted in 100 µL of bicarbonate buffer [50 mM Na2CO3 (Thermo Scientific), 50 mM NaHCO3 (Sigma-Aldrich), pH 9.6] for 1-hour incubation in high-binding 96-well plates (Biomat), whereas biotin-tagged peptides were diluted in PBS for incubation in streptavidin-coated 96-well plates (Biomat). After protein or peptide adhesion, the plates were then washed 5 times with Washing Buffer and incubated overnight with Blocking Buffer. Following another wash, wells were incubated for 1 h with 100uL of sera collected from ASFV infected domestic pigs (kindly provided by CISA-INIA), used individually or grouped into pools according to days post infection (dpi), diluted 1/200 in Blocking Buffer (Supplementary Table S7). After plate washing, wells were incubated for 1 h with 100 µL of anti-pig IgM conjugated with HRP (Bio-Rad, ref. AAI48P), diluted 1/10000 in Blocking Buffer, or anti-pig IgG conjugated with HRP (Sigma-Aldrich, ref. AP166P), diluted 1/25000. After another wash, 100 µL of TMB-E reagent (Merck) was added to each well for a 30-minute incubation, followed by 100 µL of 0.1 M H_2_SO_4_ (Merck) to stop the reaction. Absorbance was read at 450 nm, with subtraction of 550 nm absorbance for background correction. For normalization of absorbance values to 0 dpi, a minimum absorbance value of 0.050 was considered. For absolute quantification of IgG or IgM concentration, additional wells were coated with serial dilutions of pig IgM (Bio-Rad) or pig IgG (Sigma-Aldrich), blocked and incubated with anti-pig IgM HRP or anti-pig IgG HRP, respectively. GraphPad Prism, version 9.0 (GraphPad software), was used for data processing and graph design.

## Results

### ASFV-pI215L is highly conserved across genetically diverse ASFV isolates, showing evolutionary proximity to Eukarya

**Figure 1.**
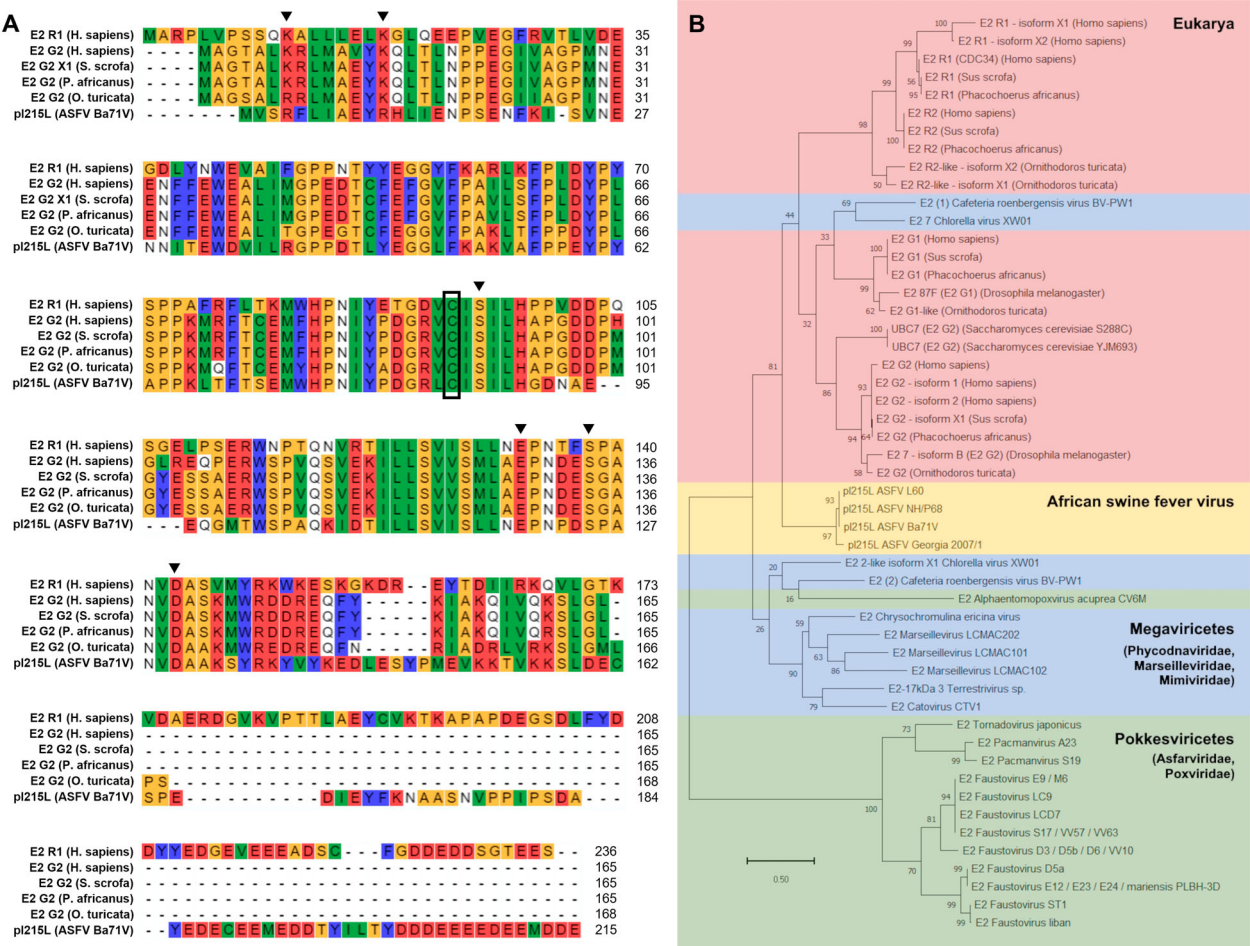
Sequence alignment of ASFV-pI215L with eukaryotic and viral ubiquitin-conjugating enzymes highlights conservation of UBC domain and suggests ASFV-pI215L evolutionary proximity to Eukarya. **(A)** Multiple sequence alignment of Ba71 V ASFV-pI215L with eukaryotic ubiquitin-conjugating proteins of high homology: E2R1 from *H. sapiens* (Cdc34), E2G2 from *H. sapiens*, *S. scrofa*, *P. africanus* and *O. turicata*. Clustal alignment: red for charged residues (negative and acidic D, E, positive and alkaline R, H, K), yellow for small residues (S, T, P, G, A), white for polar uncharged residues (N, Q), green for nonpolar aliphatic residues (C, V, I, L, M) and blue for nonpolar aromatic residues (F, Y, W). The black box indicates the ubiquitin-conjugating active site and black triangles indicate residues targeted for single-point mutagenesis. **(B)** Phylogenetic tree of a curated selection of ubiquitin-conjugating proteins: NCBI BLASTP search of proteins homologous to Ba71 V ASFV-pI215L, restricted to eukaryotic organisms and ASFV hosts (*H. sapiens*, *D. melanogaster*, *S. cerevisiae*, *S. scrofa*, *P. africanus*, *O. turicata*, in red), or non-ASFV viruses (all from *Megaviricetes* class, in blue); ASFV-pI215L sequences of genotype I and II ASFV isolates (in yellow); NCBI database and TBLASTN search of ubiquitin-conjugating proteins predicted to be encoded by non-ASFV *Asfarviridae* or *Poxviridae* (*Pokkesviricetes* class, in green). Branch length, measured in the number of substitutions per site, is indicated in the scale bar, and values next to nodes indicate bootstrap value, denoting cluster consistency (%). Accession numbers for each protein can be consulted in Supplementary Tables S1 and S2.

A phylogenetic study was first developed to assess the relationship of ASFV-pI215L to other viral and eukaryotic E2 enzymes, as well as to better understand the conservation of ASFV-pI215L across genetically diverse ASFV isolates, to estimate regions of interest for mutagenesis. First, a BLAST search was performed to identify other E2 enzymes with substantial homology to ASFV-pI215L expressed by eukaryotic organisms, including ASF hosts, and non-ASFV viruses. ASFV-pI215L shows highest similarity to the E2G2 and E2R2 enzymes expressed by ASF hosts and other Eukarya, particularly *Ornithodoros turicata*. Additionally, E2R2 and E2R1 expressed by *Homo sapiens*, *Sus scrofa* and *Phacochoerus africanus* show over 50% sequence identity with ASFV-pI215L (Supplementary Table S1), likely due to the presence of similar C-terminal acidic tails (Figure 1A). Though E2G2 enzymes present overall the highest homology to ASFV-pI215L, it is important to note that E2G2 are highly conserved among Eukarya while differing substantially from ASFV-pI215L (Figure 1A). We also found several proteins encoded by viruses other than ASFV with putative E2 activity, considerable homology to ASFV-pI215L and over 40% sequence identity, mostly encoded by viruses of the *Mimiviridae* and *Marseilleviridae* families (Supplementary Table S1). An NCBI database search provided an additional trove of putative E2 enzymes expressed by non-ASFV viruses of the *Asfarviridae* family, namely faustovirus, pacmanvirus and tornadovirus, as well as by one virus of the related *Poxviridae* family (Supplementary Table S2).

**Figure 2.**
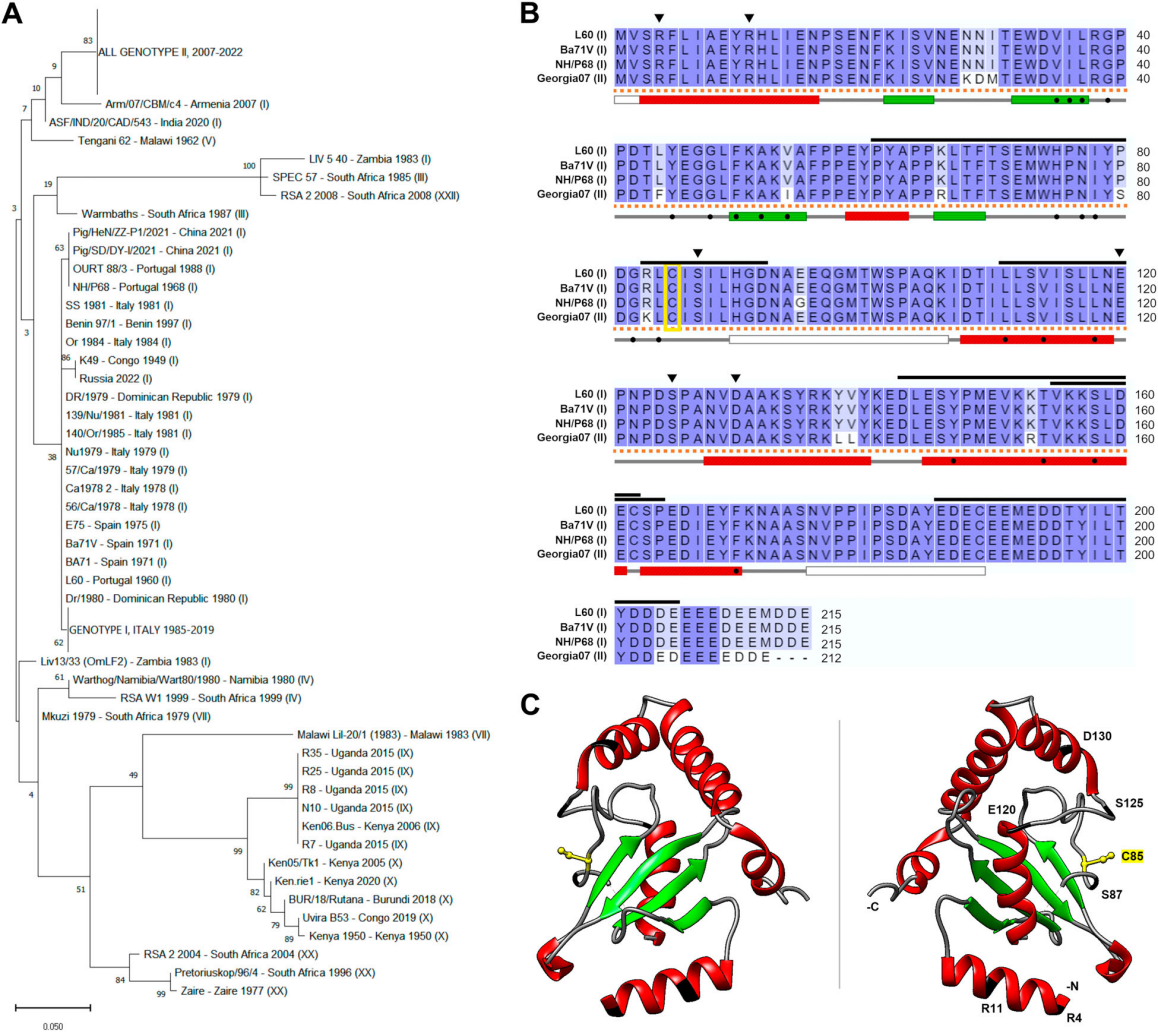
ASFV-pI215L shows high sequence conservation across genetically diverse ASFV isolates. **(A)** Phylogenetic tree of ASFV-pI215L sequences encoded by 222 ASFV genomes retrieved from the NCBI database, showing country, year of collection and, in parentheses, p72 genotype. The collapsed branch encompassing all genotype II sequences includes 116 entries from Asian and European origin, while the branch for Italy genotype I includes 60 entries. Branch length, measured in the number of substitutions per site, is indicated in the scale bar, and values next to nodes indicate bootstrap value, denoting cluster consistency (%). Accession numbers for each entry can be consulted in Supplementary Table S3. **(B)** Alignment of ASFV-pI215L sequences from the following ASFV isolates: highly virulent genotype I L60 isolate, low virulent genotype I NH/P68 isolate, avirulent genotype I Ba71 V and highly virulent genotype II Georgia 2007/1 isolate. Identical residues are shown in dark blue and the yellow box indicates ubiquitin-conjugating active site C85. Above the sequence alignment: black triangles indicate residues considered for single-point mutagenesis; black lines mark regions predicted by SVMTriP as linear B-cell epitopes, using queries of 10 or 20 residues. Below the sequence alignment: the dashed orange line highlights a 1–160 region classified as the UBC domain (Prosite ProRule PRU00388); the grey line indicates secondary structure annotated in the crystal structure of the 1–189 residue region of genotype II ASFV-pI215L (PDB ID 7WLH), with green boxes for alpha helices, red boxes for beta strands, white boxes for unmodeled regions, and black circles marking identified buried residues. (**C)** 3D representation of the ASFV-pI215L crystal structure (1-189 residues, genotype II, RCSB PDB 7WLH): the α-helix structures are shown in red, the anti-parallel sheet of β-strands in green, the C85 active site is coloured in yellow with ball and stick representation of the thiol side chain, and residues considered for single-point mutagenesis are coloured and labelled in black (right panel). The left panel shows the C85 thiol group oriented above the plane, while in the right panel the thiol group is shown below the plane.

The phylogenetic analysis of the retrieved E2 sequences showed that proteins from eukaryotic organism’s cluster according to E2 subtype and are mostly separated from viral E2 enzymes. ASFV-pI215L clusters apart from other viral E2s and appears to be more closely related to Eukarya E2s, particularly E2G2. Interestingly, putative E2 enzymes encoded by other asfarviruses seem to be more distantly related to ASFV-pI215L than those from the *Megaviricetes* class (Figure 1B). Focusing the analysis on ASFV-pI215L, 222 protein sequences were retrieved from complete ASFV genomes available at the NCBI Nucleotide database. Phylogenetic analysis of these sequences showed a genetic diversity somewhat correlated to identified ASFV genotype (Figure 2A). Remarkably, the aminoacidic sequence of ASFV-pI215L is identical in the 60 isolates collected in Sardinia since 1985 and across over 100 genotype II genomes sequenced between 2007 and 2022. Additionally, ASFV-pI215L sequences of two genotype I isolates recently collected in Asia are identical to older NH/P68 and OURT 88/3 isolates (Figure 2A). The alignment of the ASFV-pI215L sequences from ASFV isolates of genotypes I and II showed sequence identity above 90%, with genotype II differing in single residue alterations and a C-terminal deletion (Figure 2B). Recently, a resolved crystal structure of the UBC domain of genotype II ASFV-pI215L was released [30], confirming the surface exposure of the C85 active site and revealing the formation of four antiparallel sense sheets and five helix structures within the UBC domain (Figure 2B and C).

All the aforementioned data deepened our understanding of the ASFV-pI215L sequence, supporting the design of mutations in key regions of the protein to impair its enzymatic and immunogenic properties.

### Mutation of ASFV-pI215L 11–15 and 130–134 residues elicits moderate to total loss of E2 activity

**Figure 3.**
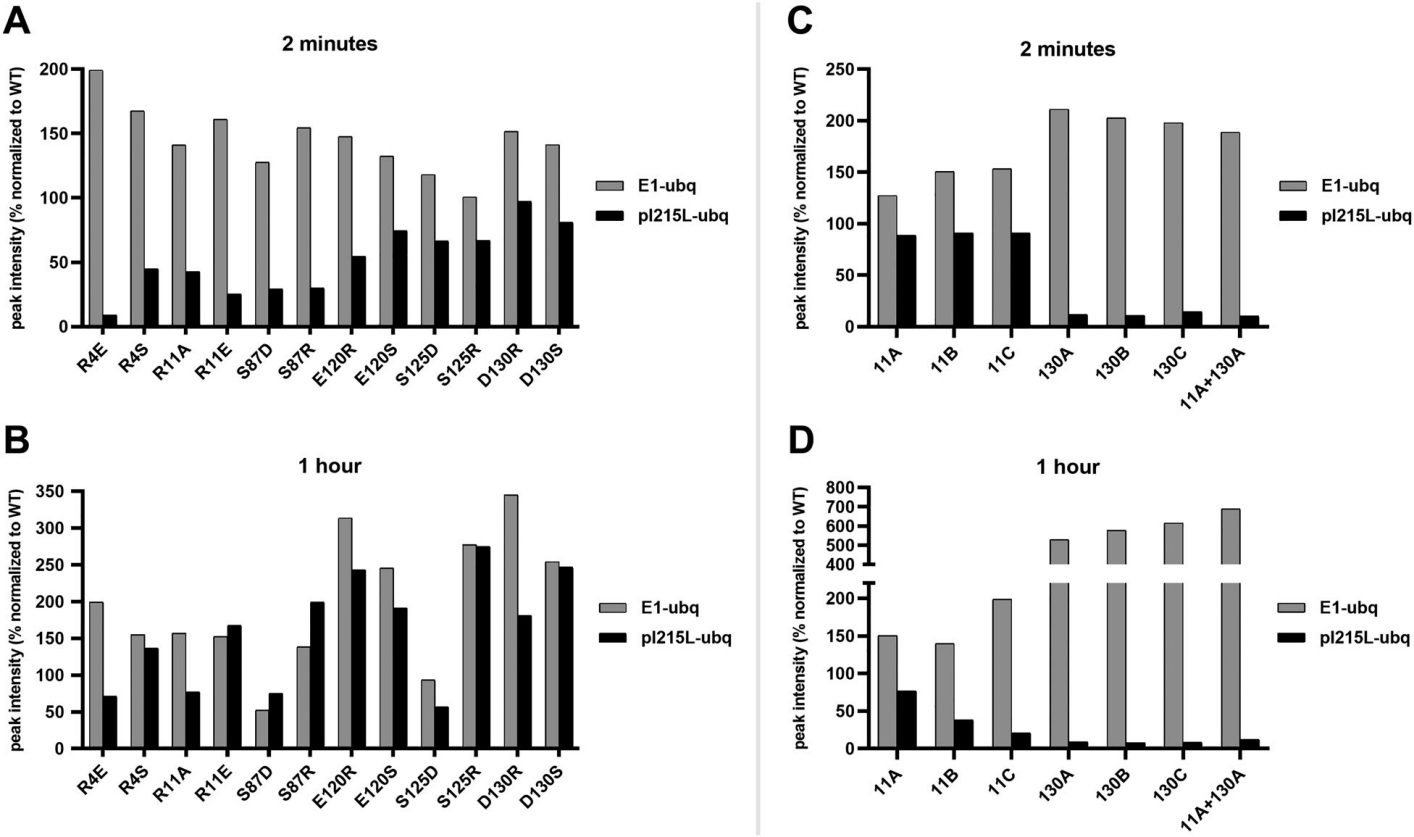
Western blotting detection of the ubiquitin-conjugating activity of ASFV-pI215L mutants showing functional impairment with single – and multi-residue substitutions in key positions of the ASFV-pI215L sequence. Graphical representation of band intensities of ubiquitin-linked protein (E1-ubq, pI215L-ubq), after incubation of ubiquitin-activating enzyme (E1), ubiquitin and ASFV-pI215L mutants, normalized to the band intensity from ASFV-pI215L^WT^. Band detection of ubiquitinated proteins can be consulted in Supplementary Figure S1. **(A)** 2-minutes ubiquitination of ASFV-pI215L single-residue mutants (R4E, R4S, R11A, R11E, S87D, S87R, E120R, E120S, S125D, S125R, D130R, D130S). **(B)** 1-hour ubiquitination of single-residue mutants. **(C)** 2-minutes ubiquitination of ASFV-pI215L multi-residue mutants (11A, 11B, 11C, 130A, 130B, 130C, 11 + 130A). **(D)** 1-hour ubiquitination of multi-residue mutants.

Based on previous mutagenesis studies involving E2 enzymes from other species, namely the human E2 enzyme Cdc34, six positions of ASFV-pI215L were selected (Figure 2C) for replacement by a residue with high or low structural similarity. After production and purification of recombinant ASFV-pI215L with the wildtype sequence (ASFV-pI215L^WT^) and twelve single-point ASFV-pI215L mutants, the functional impairment caused by each mutation was assessed by comparison to ASFV-pI215L^WT^ E2 activity: each protein was incubated at 37°C, for 2 min or 1 h, with ubiquitin, E1 and ATP, for ubiquitin activation and transference from the E1 enzyme to ASFV-pI215L, followed by Western blot detection of ubiquitin-linked protein (Figure 3A and B). For the short incubation period, mutants at residues 4 (R4E, R4S), 11 (R11A, R11E) or 87 (S87D, S87R) showed lower pI215L-ubiquitin levels, along with an accumulation of ubiquitin bound to E1, (Figure 3A and Supplementary Figure S1A). Furthermore, in the long incubation period, almost all single-point mutants at residues 120, 125 or 130 exhibited a considerable accumulation of ubiquitin conjugated with both E1 and ASFV-pI215L, suggesting that a single-residue mutation is capable of impairing ASFV-pI215L E2 activity (Figure 3B and Supplementary Figure S1B).

Then, two single-point mutations showing mild (ASFV-pI215L^R11A^) and severe (ASFV-pI215L^D130S^) impairment were used to design seven multiple-residue ASFV-pI215L mutants, replacing residues between positions 11 and 15 or 130 and 134, or both, with substitutions of three varying degrees of structural similarity (Supplementary Table S4). Notably, all ASFV-pI215L mutants with residue substitutions in the 130–134 region (130A, 130B, 130C, 11 + 130A) showed very low levels of pI215L-ubiquitin conjugates and high accumulation levels of E1-ubiquitin conjugates for both incubation periods (Figure 3C and D), revealing a severe impairment of E2 activity in mutants. However, the ASFV-pI215L mutants with substitutions in the 11–15 region showed a smaller decrease in activity (less than 20%) in the short incubation period (Figure 3C and Supplementary Figure S1C), while with longer incubation periods, their activity decreased between 20-80% (Figure 3D and Supplementary Figure S1D). Mutants with less structural similarity to ASFV-pI215L^WT^ exhibited lower E2 activity, demonstrating the functional importance of the N-terminal region despite its distance from the 85-cysteine active centre and its susceptibility to rational impairment.

### ASFV-pI215L shows an immunodominant B-cell epitope between residues 61–69 recognized by IgG and IgM from ASFV-infected pigs

**Figure 4.**
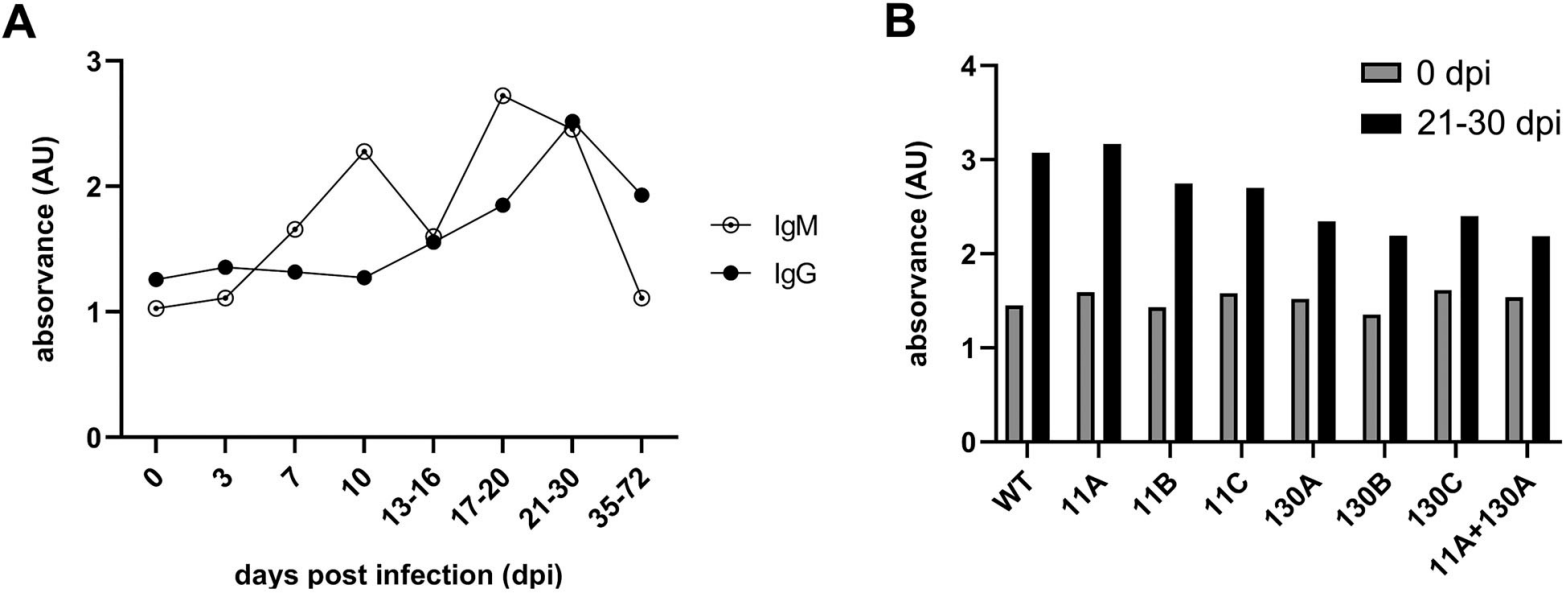
ELISA assessment of ASFV-pI215L immunogenicity after incubation of full-length recombinant ASFV-pI215L with sera collected from ASFV-infected pigs, showing late detection of IgG, early detection of IgM, and decrease of IgG recognition with analysis of multiple-residue mutations. **(A)** Absorbance detection of total IgM or total IgG after ASFV-pI215L^WT^ incubation with eight sera pools ranging from 0 to 35-72 dpi. **(B)** ASFV-pI215L^WT^ and eight multi-point ASFV-pI215L mutants incubated with sera pool from 0 or 21-30 dpi, for detection of total IgG with antigen affinity to ASFV-pI215L.

The recognition of ASFV-pI215L by ASFV-related immunoglobulins was evaluated by incubation of the full-length protein with pools of sera from ASFV infected pigs (Supplementary Table S7), to compare total IgG or IgM levels bound to ASFV-pI215L throughout the infection. An increase of IgG levels was observed from 13-16 dpi onwards, reaching its maximum at 21-30 dpi, while IgM levels were found to be increased from 7 dpi, showing two peaks at 10 dpi and 17-30 dpi (Figure 4A). Comparing IgG recognition of ASFV-pI215L^WT^ and multi-point mutants, a slight reduction of IgG levels at 21-30 dpi was detected in the latter, particularly in proteins with mutations in the 130–134 region (Figure 4B), suggesting that mutagenesis of the ASFV-pI215L sequence may suppress epitope recognition.

**Figure 5.**
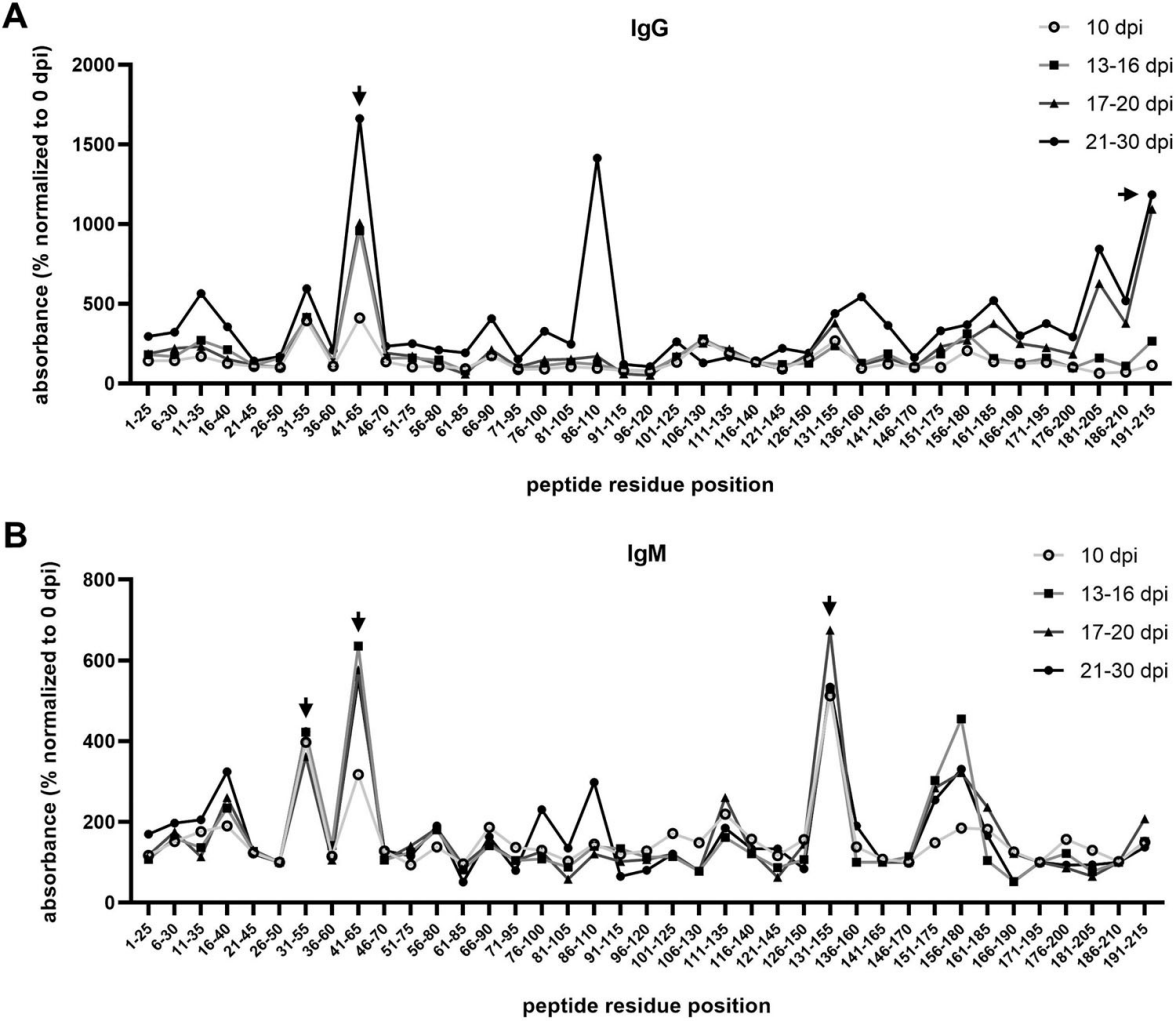
ELISA quantification of total IgG and IgM in sera from ASFV-infected pigs, grouped according to dpi, with affinity to the biotin-tagged ASFV-pI215L peptide library (25-residue length), for selection of ASFV-pI215L regions of interest highly recognized by ASFV-related immunoglobulins (marked with black arrows). **(A)** Detection of total IgG in each sera pool binding to each peptide, normalized to absorbance measurement with 0 dpi pool (n = 3). **(B)** Detection of total IgM in sera binding to each peptide, normalized to 0 dpi (n = 3). A graphical representation of the measured absorbance units for IgG and IgM detection, without normalization to 0 dpi, can be consulted in Supplementary Figure S2.

The use of full-length protein for antibody capture was followed with a library of 39 biotin-tagged peptides, each comprising sequential 25-residue segments of ASFV-pI215L (Supplementary Table S5). The procedure aimed to map linear B-cell epitopes. For IgG, we found that the best suited peptides corresponded to the 41–65 region and the C-terminal tail within position 181-215, with recognition beginning at the second week of infection (Figure 5A, Supplementary Figure S2). The 86–110 region was relevant but not considered further given its proximity to the C85 active site. Regions 31–65 and 131–155 seemed to have the highest affinity to ASFV-related IgM, as early as 7 days after infection (Figure 5B, Supplementary Figure S2).

**Figure 6.**
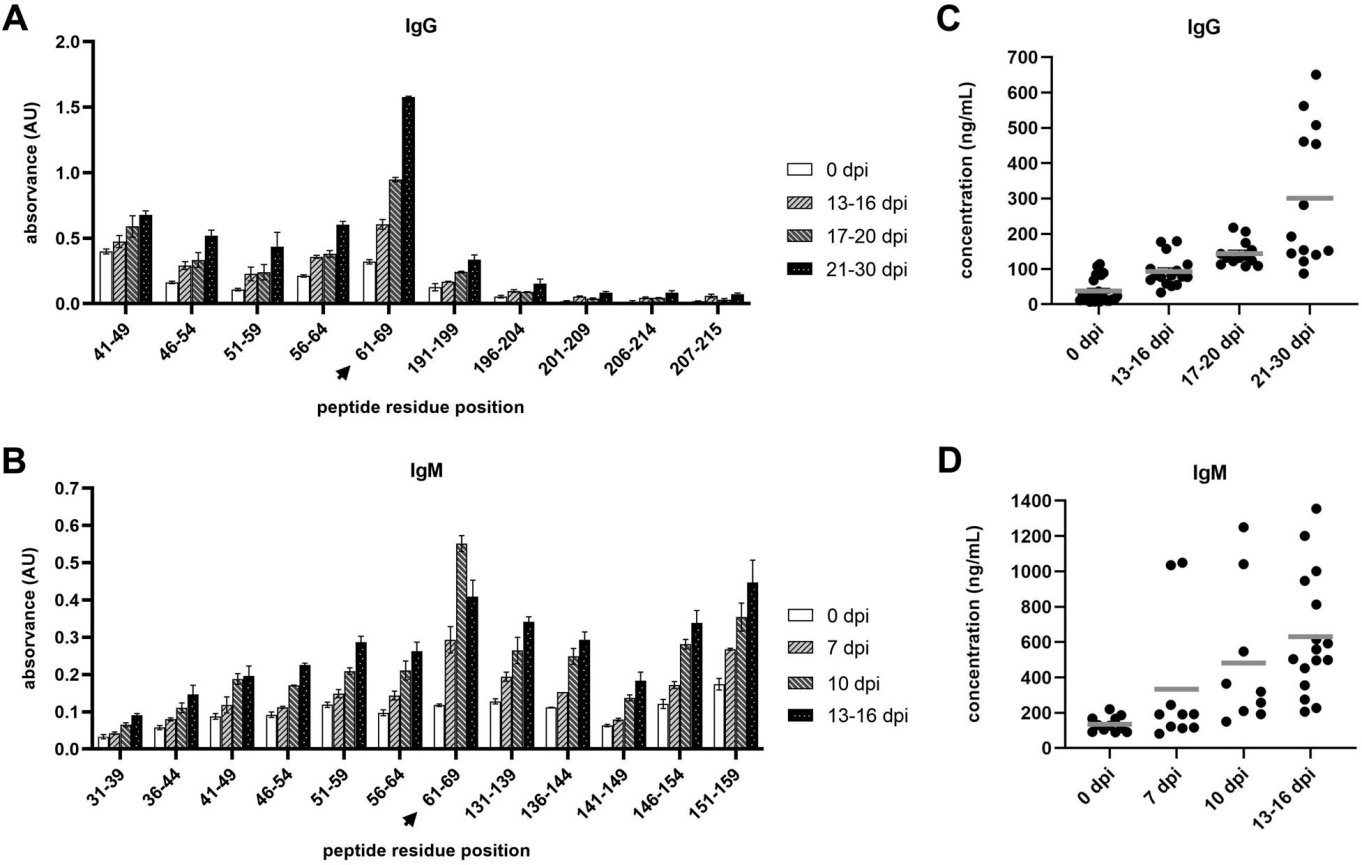
ELISA quantification of immunoglobulin affinity of ASFV-infected sera to the PS-tagged ASFV-pI215L peptide library (9-residue length) suggests that the ASFV-pI215L 61–69 region, marked with a black arrow, contains a B-cell epitope recognized by ASFV-related IgG and IgM. **(A)** Absorbance measurement of total IgG after incubation of selected PS-tagged peptides with pools of sera from ASFV-infected pigs, grouped according to dpi (n = 3). **(B)** Absorbance measurement of total IgM after incubation of selected PS-tagged peptides with sera pools (n = 3). **(C)** Quantification of total IgG with affinity to PS-tagged peptide of the ASFV-pI215L 61–69 region, after incubation with individual sera from 0 and 13–30 dpi (n = 3) **(D)** Quantification of total IgM with affinity to the 61–69 region peptide, using individual sera from 0 and 7–16 dpi (n = 3).

The screening for ASFV related epitopes was further narrowed with the use of a library of PS-tagged peptides of 9 residues each (Supplementary Table S5), restricted to the ASFV-pI215L regions of interest suggested in the previous assay. Pools of sera from early – or late-stage infections were used for IgM and IgG analysis, respectively. Notably, the same peptide ranging from residues 61–69 showed the highest affinity to both ASFV-related IgG (Figure 6A) and IgM antibodies (Figure 6B). Then, the immunogenicity of the ASFV-pI215L 61–69 region was further assessed by incubating the peptide with individual sera, showing a considerable increase of IgG and IgM recognition throughout the disease course (Figure 6C and D).

### Multiple-residue mutations in the 62–69 region suppress antibody recognition and reduce ASFV-pI215L enzymatic activity

**Figure 7.**
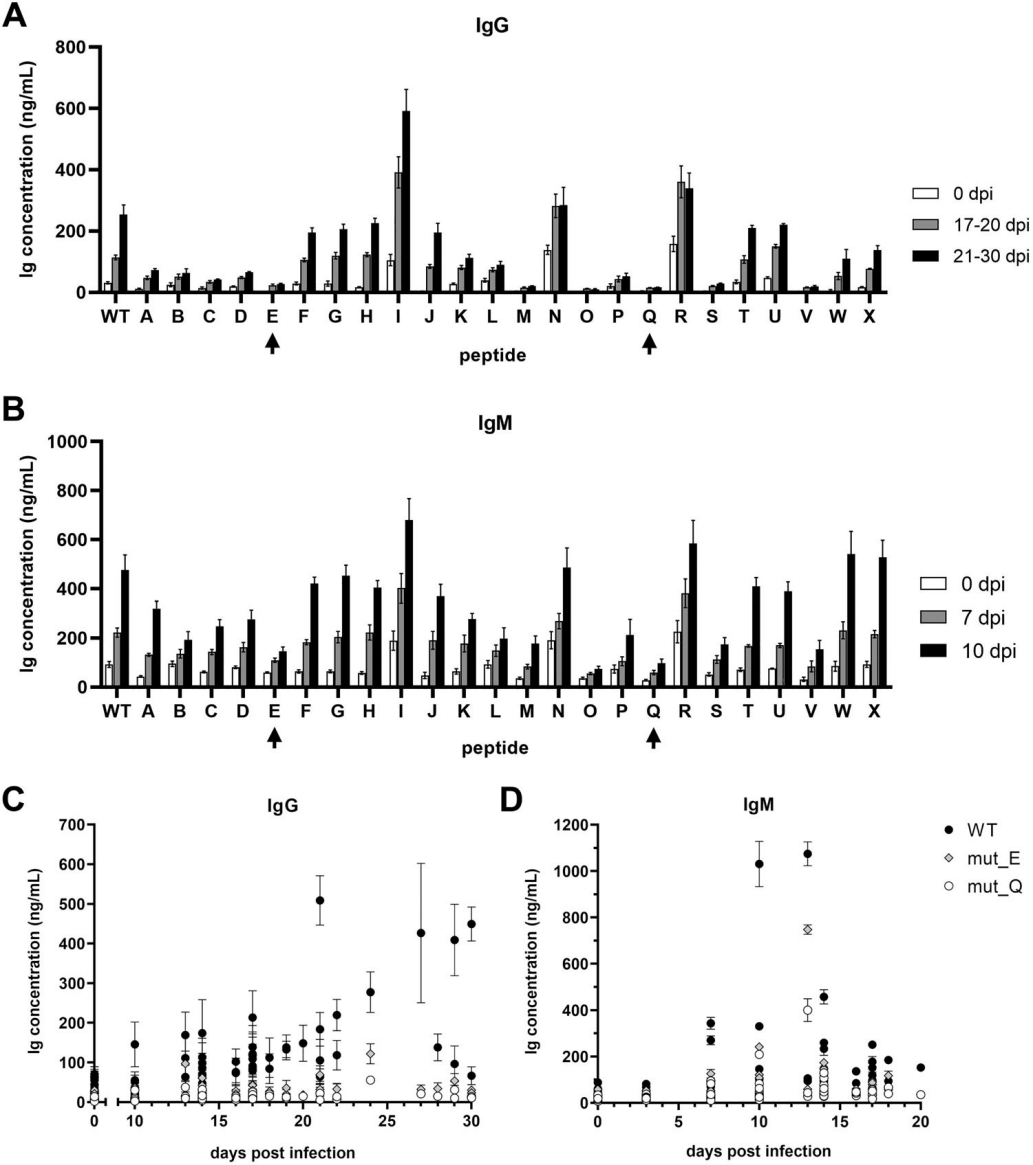
ELISA screening of immunoglobulin affinity of ASFV-infected sera to the PS-tagged peptide library containing ASFV-pI215L 61–69 wildtype (WT) and mutant sequences (A to X), with mutant peptides E and Q presenting a significant decrease in recognition by both IgG and IgM. **(A)** Quantification of total IgG after incubation of wildtype and mutant sequence peptides with pools of sera from ASFV-infected pigs, grouped according to dpi (n = 3). **(B)** Quantification of total IgM after incubation of wildtype and mutant sequence peptides with sera pools (n = 3). **(C)** Incubation of peptide of the ASFV-pI215L 61–69 region wildtype sequence (WT), or selected mutants E and Q, with individual sera from 0 and 10–30 dpi for quantification of total IgG with antigen affinity to peptide (n = 3) **(D)** ASFV-pI215L 61–69 wildtype (WT) or selected mutants E and Q incubated with individual sera from 0 to 20 dpi for quantification of total IgM (n = 3). A more comprehensive graphical representation of quantified IgG and IgM concentration after peptide incubation with individual sera, sorted by sera sample and grouped according to each infected animal, can be consulted in Supplementary Figure S3.

After identifying the immunogenic potential of the ASFV-pI215L 61–69 region (PYAPPKLTF), we needed to assess which sequence alteration could diminish epitope recognition and affinity to ASFV-related IgM and IgG, for which it was designed mutant peptides of decreasing similarity to the wildtype 9-residue sequence (Supplementary Table S6). Two peptides displayed noticeably lower levels of IgG and IgM affinity compared to the wildtype sequence: peptide E (PEDPPKLTF) with substitutions at positions 62 and 63, and peptide Q (PNSPPQTLQ) with six residue substitutions at positions 62, 63 and 66- 69 (Figure 7A and B). To evaluate the reduction in immunogenicity, the selected peptides were also incubated with individual sera, confirming the broad recognition of the ASFV-pI215L 61–69 region by ASFV-related IgM and IgG, and the reduced affinity of both mutant sequences (Figure 7C and D, Supplementary Figure S3). It was particularly pronounced with mutant sequence Q, which completely abrogated recognition by IgG in almost all tested sera.

**Figure 8.**
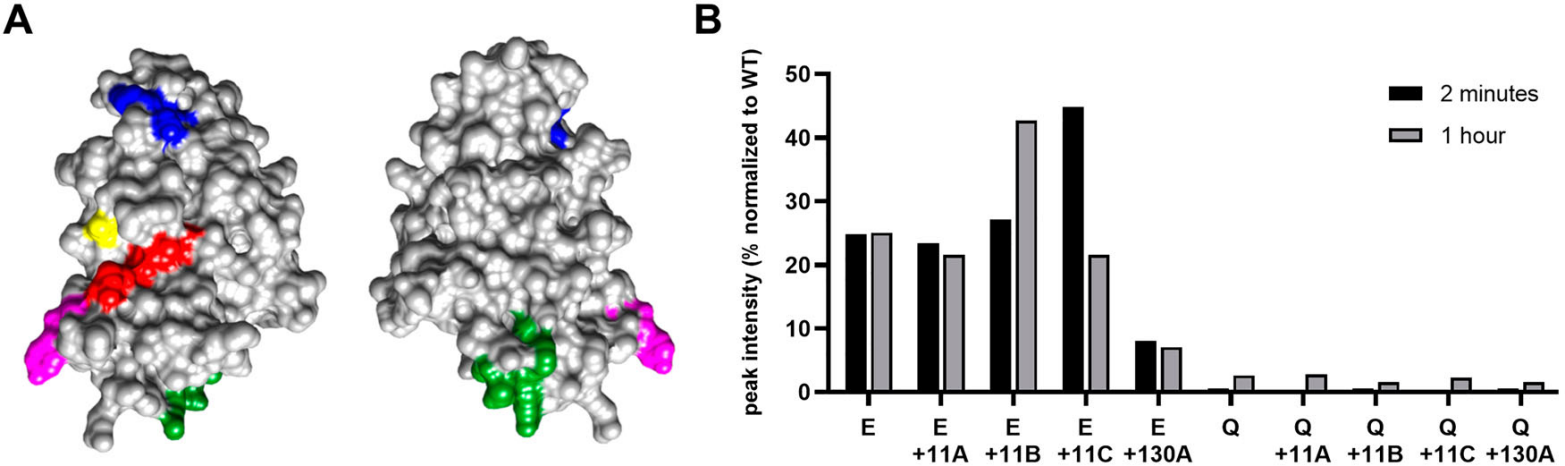
Structure and function analysis of mutant ASFV-pI215L containing residue substitutions for suppressing epitope recognition and further impairing ubiquitin-conjugating activity, with placement of epitope mutation E showing severe impairment of ubiquitin-conjugating capacity and mutation Q resulting in loss of function. **(A)** Front and back view of a 3D representation of the protein surface of the ASFV-pI215L 1–189 residue region (RCSB PDB 7WLH), showing the 85-cysteine active centre in yellow, the 11–15 residue region targeted for functional mutations (11A, 11B, 11C) in green, the 130–134 residue region targeted for functional mutation (130A) in blue, residues 62 and 63 altered in epitope mutation E and mutation Q shown in pink, and the 66–69 residue region additionally altered in epitope mutation Q shown in red. **(B)** Graphical representation of the western blot band intensities of pI215L-ubq conjugates after 2-minutes or 1-hour ubiquitination of ASFV-pI215L epitope mutants (full-length protein containing the epitope mutation E or Q alone or combined with functional mutations 11A,11B, 11C or 130A), normalized to the band intensity from ASFV-pI215L^WT^. Band detection of ubiquitinated proteins can be consulted in Supplementary Figure S1.

Incorporating the ASFV-pI215L 61–69 region mutations into the viral genome could potentially reduce ASFV-pI215L immunogenicity and prompt an altered immune response during infection, but sequence alterations in an area within such close proximity to the C85 active centre could also influence ASFV-pI215L’s E2 activity (Figure 8A). To verify this, it was designed a new set of recombinant ASFV-pI215L mutants containing epitope suppressing mutations E or Q, alone or in combination with previously tested functional mutations, in an attempt to obtain a mutant sequence for ASFV-pI215L that could impair its E2 activity and alter immunoglobulin affinity. Insertion of the epitope mutation E in ASFV-pI215L resulted in a protein with diminished E2 activity, presenting 20% of its ubiquitin intake capacity in comparison to ASFV-pI215L^WT^, while a larger decrease was observed in the mutant pI215L combining mutation E with functional mutation 130A. The Q epitope mutation appeared to result in almost total E2 loss of function of the full-length protein, either when alone or when combined with other mutations affecting function (Figure 8B and Supplementary Figure S1E and S1F).

## Discussion

ASFV genome modification, based on the alteration or removal of genes with either undisclosed function or known to be relevant for viral infection and immunosuppression, has become a standard procedure in ASFV vaccine research [1,31,32]. Recently, ASFV-pI215L has been shown to have a key role in the ASFV infectious cycle and IFN-I inhibition, suggesting it as a promising target for the development of live vaccine candidates [33]. Indeed, the rational mutagenesis of ASFV-pI215L towards the decrease of E2 activity could have effects in ASFV pathogenicity and might result in a recombinant virus with reduced virulence. This rationale guided our research strategy to deepen the knowledge of the enzymatic and immunogenic properties of ASFV-pI215L.

A phylogenetic analysis of ASFV-pI215L was firstly envisioned to understand its conservation among ASFV isolates and its evolutionary relationship with viral and eukaryotic E2 enzymes, akin to other comparative studies [34,35]. ASFV has long been reported to be the only virus expressing a protein with sequence homology to E2 enzymes and confirmed E2 activity [9,14,15,17], but recent genome sequencing of large viruses allowed for the identification of other proteins with putative E2 activity and also substantial homology with ASFV-pI215L (Supplementary Tables S1 and S2). Homologous viral E2 enzymes are encoded by viruses of the *Megaviricetes* class, most of them belonging to *Mimiviridae* and *Marseilleviridae* families and some encoding up to five E2 enzymes. Within the *Asfarviridae* family, E2 enzymes are encoded by faustovirus and pacmanvirus [36,37], though other members such as the abalone asfa-like virus and kaumoebavirus do not seem to express any. One virus of the *Poxviridae* family encodes a E2 enzyme, which composes the *Pokkesviricetes* virus class along with *Asfarviridae*, and both *Megaviricetes* and *Pokkesviricetes* classes constitute the *Nucleocytoviricota* phylum that comprise the nucleocytoplasmic large DNA viruses (NCLDV) [38], but putative E2 enzymes have also been reported in non-NCLDV [39–41].

The phylogenetic tree generated with eukaryotic and viral ubiquitin-conjugating proteins (Figure 1B) shows a clustering of E2 enzymes according to Eukarya, ASFV, *Megaviricetes* or *Pokkesviricetes* origin. Notably, ASFV-pI215L was grouped apart from other *Asfarviridae* E2 enzymes and showed more evolutionary proximity to eukaryotic proteins, particularly with E2G2. Furthermore, the genomic encoding of an E2 enzyme is not conserved within the *Asfarviridae* family, suggesting that ASFV-pI215L has a distinct origin from other asfarviruses, and could share a common ancestor with Eukarya E2 enzymes. In addition, current genome sequencing efforts have revealed significant insights into the epidemiologic progression of genotype I and II ASFV outbreaks [42,43]. In this study, more than two hundred ASFV-pI215L amino acidic sequences were used to generate a phylogenetic tree, which shows a similar pattern to ASFV-p72 and a low mutation rate (Figure 2A). Despite some sequence variation in genotype I isolates collected from the first outbreaks in Europe, ASFV-pI215L is fully conserved within all genotype I isolates from Sardinia, as well as all queried sequences of genotype II viruses. Furthermore, ASFV-p215L sequences of genotype I isolates recently collected in Asia are identical to former European isolates NH/P68 and OURT 88/3, as previously reported for other viral genes [44–46]. Notably, the ASFV-p215L sequence from genotype I NH/P68 shows 90% identity when compared to genotype II isolates, demonstrating high conservation of the UBC domain and presumably E2 activity. In sum, the ASFV-pI215L is a unique protein showing a distinct origin and a conserved sequence within diverse ASFV isolates, especially in circulating genotypes I and II.

Regarding the mutagenesis studies, twelve recombinant single-point ASFV-pI215L mutants were designed, targeting conserved residues in ASFV genotypes I and II, as well as in Eukarya E2R1 and E2G2 (Figure 1A), and considering previous reports on the human E2R1 [47–52]. Following protein production, the E2 activity of ASFV-pI215L mutants was then evaluated and compared with ASFV-pI215L^WT^, particularly regarding the diminished capacity to receive ubiquitin transferred from an E1 enzyme (Figure 3A and B). Two single-point mutants (ASFV-pI215L^R11A^; ASFV-pI215L^D130S^) were selected to further generate multiple-residue mutants, due to their mild and severe hampering of E2 activity. Whereas the R11 residue is located in the α1-helix (Figure 2C), at the N-terminus of the UBC domain, an area known to contain arginine residues involved with E1-E2 interaction and thioester activity, whereas the D130 residue locates in the α4-helix at the C-terminus of the UBC domain (Figure 2C), a region that accommodates and interacts with the negatively-charged and C85-linked ubiquitin [48,50].

Despite the low mutation rate of ASFV, the development of vaccines based on multiple-residue mutants is pointed to increase its biosafety by preventing spontaneous reversion [53]. Thus, we also designed and produced seven multiple-residue mutants by substituting at least five consecutive residues. Our results showed that ASFV-pI215L mutants with substitutions in residues 11–15 showed a slight decrease in enzymatic activity during short-term incubation, while during long-term incubation, the more the sequence diverged from ASFV-pI215L^WT^ the greater the reduction in E2 activity was observed (Figure 3C and D). All mutants with residue substitution of the 130–134 region showed over 80% decrease of ubiquitin binding with an accumulation of E1-ubiquitin complexes, suggesting that the modification of those residues may have resulted in a conformational change which disrupts the ubiquitin transfer from E1 to ASFV-pI215L (Figure 3C and D). The above results open new insights into the ability to manipulate ASFV-pI215L enzymatic activity towards the design of recombinant ASFV with reduced E2 activity.

Besides having a crucial role in IFN-I innate antiviral response, it has been observed that ASFV-pI215L may also have the capacity to induce B-cell antibody production in immunized pigs [27]. To confirm the immunogenicity of ASFV-pI215L, the full-length recombinant wildtype ASFV-pI215L was used as antigen in ELISA assays against sera collected from pigs experimentally inoculated through different routes with different virus isolates. The production of IgG and IgM antibodies recognizing ASFV-pI215L was confirmed in the sera from all infected animals. An increase of IgM recognition was initially detected at 7 dpi, while IgG recognition started at 14-17 dpi lasting at least a month after infection (Figure 4A). Though the mechanisms responsible for animal immunization against ASF remain largely undisclosed, the production of antibodies specific to ASFV proteins has been highlighted in surviving animals infected with attenuated isolates, or challenged with virulent ASFV after immunization with gene-deleted vaccine candidates [54–56]. ASFV-pI215L could potentially be involved in this antibody-mediated protection, given the production of IgG and IgM targeting the viral protein. Moreover, the replacement of AFSV-pI215L^WT^ with multi-residue mutants, particularly the ones affecting the 130–134 region, resulted in a decrease to IgG-ASFV-pI215L binding (Figure 4B), suggesting that ASFV-pI215L mutagenesis can be instrumentalized to modulate immune recognition and to design vaccines with DIVA characteristics against ASF.

After confirming the production of IgG and IgM antibodies specific to ASFV-pI215L, sera from the above mentioned ASFV infected pigs were used to identify immunodominant B-cell epitopes within the ASFV-pI215L protein sequence, using two peptide libraries comprising segments of different size. Recently, we used this methodology to successfully report two dominant linear B-cell epitopes at bottom of the β-strand of DNA binding region in ASFV-A104R protein [35]. In our study, the 25-residue library allowed for the screening of linear epitopes encompassing the totality of the ASFV-pI215L sequence (Figure 5), from which we identified regions then subjected to a fine-tuned screening using the 9-residue peptide library. Notably, we found that the 61–69 region of ASFV-pI215L (PYAPPKLTF) showed the highest affinity to both IgG and IgM from sera of infected pigs (Figure 6), strongly suggesting that this region contains a linear antigenic determinant, potentially susceptible to manipulation for DIVA vaccine design. This confirmed the bioinformatic analysis which had predicted a B-cell epitope in this region of ASFV-pI215L (Figure 2B). A similar study found that the 67–80 and 167–176 regions of ASFV-pI215L are recognized by anti-pI215L IgG1 and IgG2a monoclonal antibodies, with the deletion of the 67–80 epitope resulting in loss of IgG recognition [57].

Following the identification of this B-cell epitope, we designed a third library of peptides with different mutations in the 61–69 region of ASFV-pI215L, with an increasing number of altered residues and decreasing residue similarity, in order to find the sequences most capable of abolishing IgG and IgM recognition. Mutations E (PEDPPKLTF) and Q (PNSPPQTLQ) were selected as the best suited to abolish recognition by both anti-ASFV IgM and IgG, with mutation Q showing particularly good results, nullifying peptide antigenicity for almost all ASFV positive sera (Figure 7). The two-residue substitution in mutation E is located at the end of the α2-helix, at a turning point exposed on the surface of ASFV-pI215L. In comparison, mutation Q consists of a six-residue substitution, the two substitutions of mutation E and four downstream, also localized at the ASFV-pI215L surface. The four downstream substitutions are localized at the β4-strand of the antiparallel sheet that characterizes the ASFV-pI215L secondary structure, and despite some linear distance from the E2 active site cysteine-85, they are spatially close (Figure 8A). Mutations in this location could seriously compromise the ASFV-pI215L E2 activity, hence another set of recombinant ASFV-pI215L mutants was produced incorporating epitope-suppressing mutations E and Q, both alone or in combination with the previously designed functional mutations at the 11–15 or 130–134 region, given that the inclusion of both types of mutations in the ORF I215L could have a synergistic or antagonistic effect over E2 activity. Our results revealed that the recombinant ASFV-pI215L containing epitope mutation E presented variable impairment of E2 activity (Figure 8B): (i) ASFV-pI215L carrying mutation E or mutations E plus 11A showed an 80% decrease of E2 activity; (ii) despite both 11B and 11C mutations having less residue similarity to the original sequence than 11A, the combination of mutations E with 11B or 11C provided less reduction in E2 activity; (iii) the combination of mutations E with 130A further decreased E2 activity to less than 10% of what was observed for ASFV-pI215L^WT^. On the other hand, all recombinant ASFV-pI215L proteins displaying mutation Q had no E2 activity. This apparent loss of function may be explained by the proximity of mutation Q to the E2 active site, and the drastic conformational changes possibly induced after total residue replacement of the β4-strand.

In sum, our results suggest that the rational mutagenesis of ASFV-pI215L may result in varying degrees of impairment of viral E2 activity for a controlled attenuation of ASFV virulence, providing an efficient strategy for the design of a live attenuated vaccine. While inactivated virus does not replicate nor provide protection, and proposed subunit or DNA-based vaccines prompt an immune response that is insufficient to protect against virulent ASFV, live attenuated viruses can provoke a broad cellular and humoral response given its replicative capacity and have shown more promising protective properties, though prevailing some issues regarding cross-protection and virus shedding. Recent manipulation of virulence-related genes has provided a more rational and safe method for further attenuation of ASF viruses, as opposed to the accumulation of non-specific mutations after multiple *in vitro* passages of field isolates, more prone to residual virulence or loss of protective capabilities [1]. Mutation-based attenuation can also be used for the implementation of a DIVA strategy, since the disrupted B-cell epitopes of immunogenic viral proteins may be repurposed as negative DIVA markers in serological tests [32]. This methodology has been applied to previous vaccine candidates [58,59], and it could be extended to ASFV-pI215L. Genome insertion of the identified functional and epitope mutations could be sufficient for virus attenuation, though it should be assessed the genetic stability and protection capacity of such vaccine prototypes, coupled with validation of complementary DIVA testing. Nevertheless, we believe our work accomplishes the purpose of providing a new perspective for the development of live attenuated DIVA vaccines, based on site-specific reduction of ASFV-pI215L E2 activity and immunogenicity.

## Supplementary Material

Figure S3B.jpg

Figure S3A.jpg

Figure S2.jpg

Figure S1.jpg

SUPPLEMENTARY TABLES.docx
