# Supplementary figures and images for: Multiple directed mutagenesis reduces enzymatic activity and antibody recognition of the African Swine Fever Virus E2 ubiquitin-conjugating protein (ASFV-pI215L)
Source: Emerg Microbes Infect. 2026 Jan 23;15(1):2622218. doi: 10.1080/22221751.2026.2622218 (PMC12885014; doi:10.1080/22221751.2026.2622218)

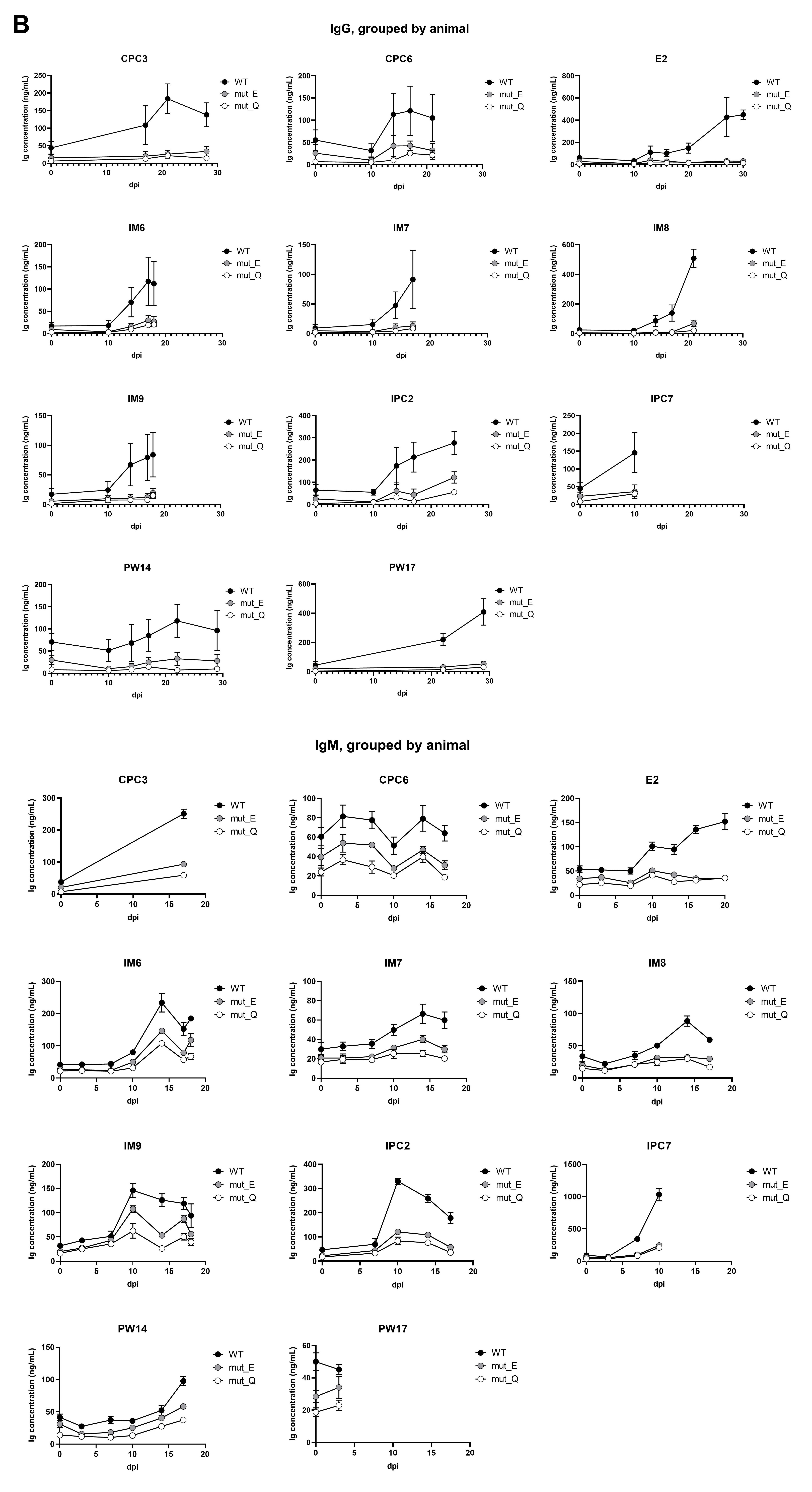

Supplement: Figure S3B.jpg [file TEMI_A_2622218_SM0390.jpg]

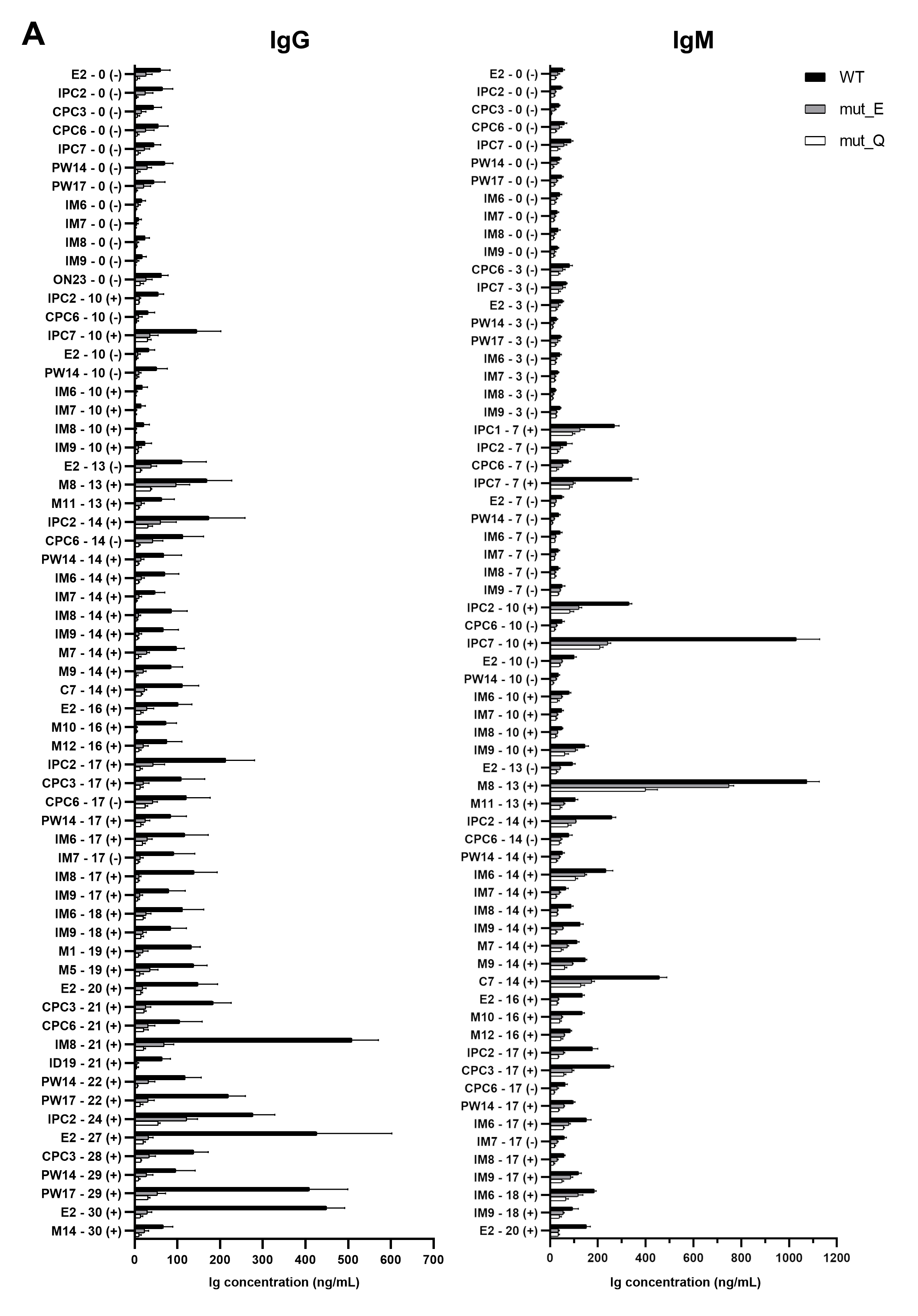

Supplement: Figure S3A.jpg [file TEMI_A_2622218_SM0389.jpg]

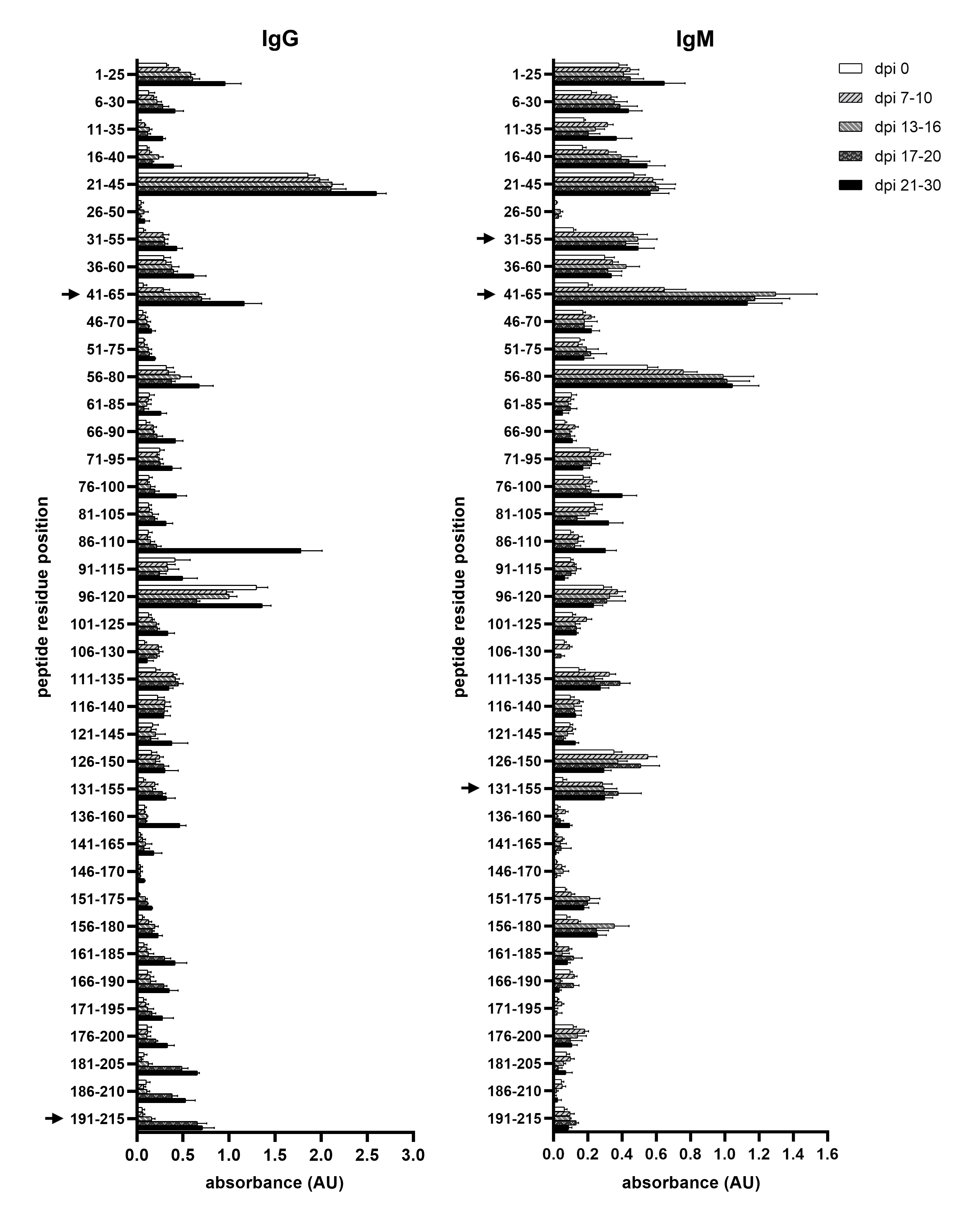

Supplement: Figure S2.jpg [file TEMI_A_2622218_SM0388.jpg]

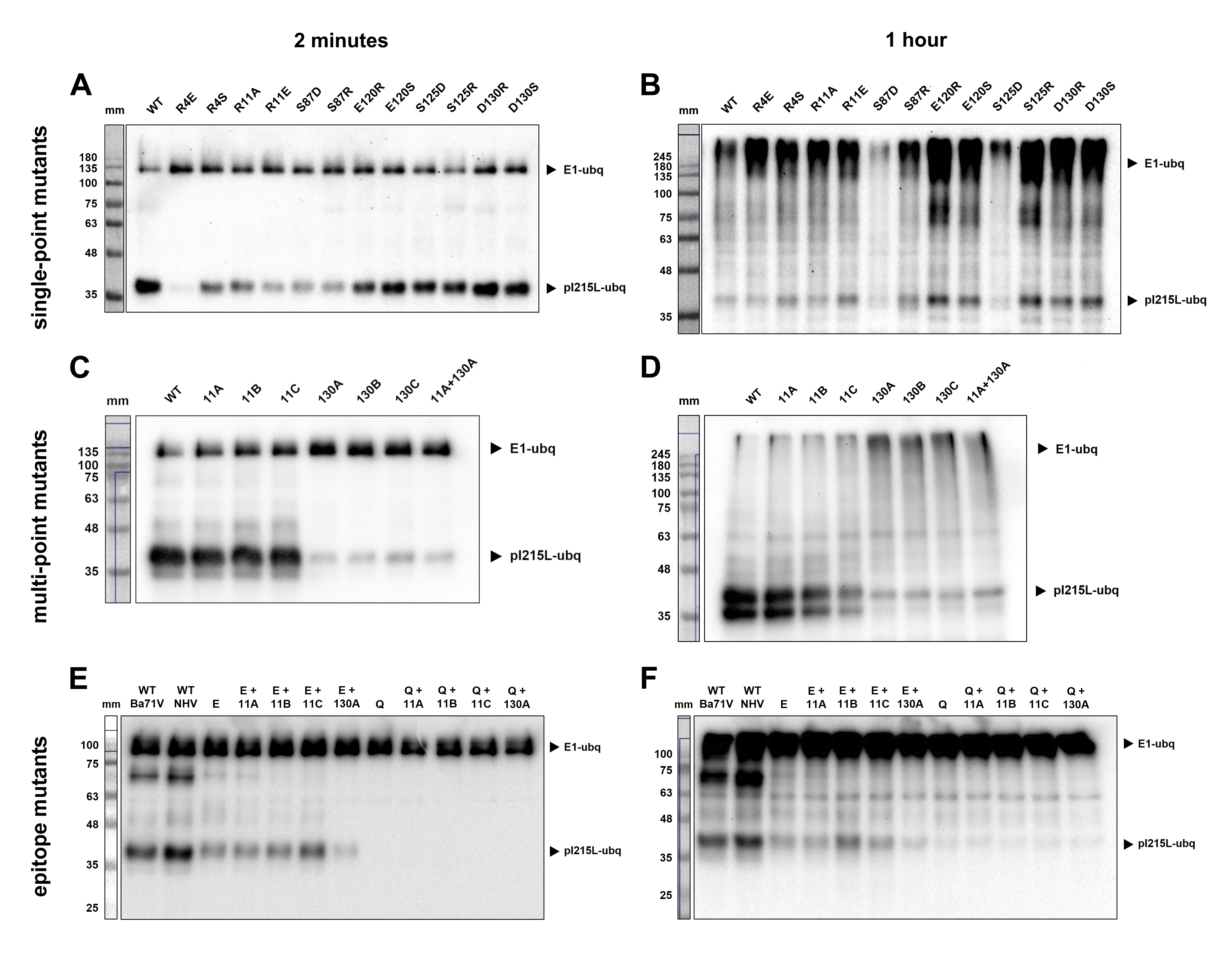

Supplement: Figure S1.jpg [file TEMI_A_2622218_SM0387.jpg]
