# Supplementary material for: Multiple directed mutagenesis reduces enzymatic activity and antibody recognition of the African Swine Fever Virus E2 ubiquitin-conjugating protein (ASFV-pI215L)
Source: Emerg Microbes Infect. 2026 Jan 23;15(1):2622218. doi: 10.1080/22221751.2026.2622218 (PMC12885014; doi:10.1080/22221751.2026.2622218)
Supplement: SUPPLEMENTARY TABLES.docx [file TEMI_A_2622218_SM0386.docx]

**SUPPLEMENTARY DATA (TABLES)**

**Table S1 -** Curated selection of ubiquitin-conjugating protein sequences, resulting from a BLASTP search with Ba71V ASFV-pI215L (Uniprot accession P27949, NCBI Protein accession AAA65370.1) as the query sequence, default settings, restricted to a selection of organisms. An e-value threshold below 1×10^-40^ was established for ASFV hosts *Sus scrofa* (taxid 9823), *Phacochoerus africanus* (taxid 41426) and Ornithodoros (taxid 6937), below 1×10^-40^ for Eukarya models *Homo sapiens* (taxid 9606) and *Drosophila melanogaster* (taxid 7227), bellow 1×10^-37^ for *Saccaromyces cerevisiae* (taxid 4932), and below 5×10-^23^ for viruses (taxid 10239) excluding African Swine Fever virus (taxid 10497). The BLASTP search restricted to ASFV host *Potamochoerus larvatus* (taxid:273792) did not provide any results, and the search restricted to Ornithodoros only provided results for *O. turicata* and did not provide any protein sequence from *O. moubata*, *verrucosus* or *erraticus* species at the time of search (July 2024). A total of 24 Eukarya and 10 viral ubiquitin-conjugating proteins were retrieved, after further exclusion of sequences from PDB crystal structures, partial proteins or proteins not annotated as E2 or ubiquitin-conjugating. Regarding the BLASTP results for non-ASFV virus, the parenthesis indicates virus isolate source and location, and the asterisk refers to second ubiquitin-conjugating proteins similar to ASFV-pI215L found in previously referred virus isolates.

| ASFV hosts (Eukarya) | | | | | | | | |
| --- | --- | --- | --- | --- | --- | --- | --- | --- |
| accession nr | **accession description** | **organism** | **family** | **max/total score** | **query cover** | **E-value** | **% identity** | **nr. res.** |
| XP_003359057.1 | ubiquitin-conjugating enzyme E2 G2 isoform X1 | Sus scrofa | Suidae | 149 | 71% | 3E-45 | 49,38% | 165 |
| XP_020920359.1 | ubiquitin-conjugating enzyme E2 R2 | Sus scrofa | Suidae | 150 | 62% | 1E-44 | 51,77% | 238 |
| XP_020939965.1 | ubiquitin-conjugating enzyme E2 R1 | Sus scrofa | Suidae | 147 | 62% | 7E-44 | 51,06% | 234 |
| XP_020923359.1 | ubiquitin-conjugating enzyme E2 G1 | Sus scrofa | Suidae | 140 | 71% | 1E-41 | 43,75% | 170 |
| XP_047653947.1 | ubiquitin-conjugating enzyme E2 G2 | Phacochoerus africanus | Suidae | 148 | 71% | 4E-45 | 48,75% | 165 |
| XP_047610455.1 | ubiquitin-conjugating enzyme E2 R2 | Phacochoerus africanus | Suidae | 150 | 62% | 8E-45 | 51,77% | 238 |
| XP_047633788.1 | ubiquitin-conjugating enzyme E2 R1 | Phacochoerus africanus | Suidae | 147 | 62% | 5E-44 | 51,06% | 234 |
| XP_047612908.1 | ubiquitin-conjugating enzyme E2 G1 | Phacochoerus africanus | Suidae | 140 | 71% | 1E-41 | 43,75% | 170 |
| XP_064474536.1 | ubiquitin-conjugating enzyme E2  R2-like  isoform X2 | Ornithodoros turicata | Argasidae | 159 | 62 | 9E-49 | 53,9 | 219 |
| XP_064473277.1 | ubiquitin-conjugating enzyme E2 G2 | Ornithodoros turicata | Argasidae | 157 | 73 | 1E-48 | 50,31 | 168 |
| XP_064474535.1 | ubiquitin-conjugating enzyme E2  R2-like  isoform X1 | Ornithodoros turicata | Argasidae | 159 | 72 | 3E-48 | 48,77 | 253 |
| XP_064460334.1 | ubiquitin-conjugating enzyme E2  G1-like | Ornithodoros turicata | Argasidae | 148 | 73 | 2E-45 | 46,95 | 167 |
| other Eukarya | | | | | | | | |
| accession nr | **accession description** | **organism** | **family** | **max/total score** | **query cover** | **E-value** | **% identity** | **nr. res.** |
| NP_003334.2 | ubiquitin-conjugating enzyme E2 G2 isoform 1 | Homo sapiens | Hominidae | 149 | 71% | 2E-44 | 49.38% | 165 |
| NP_060281.2 | ubiquitin-conjugating enzyme E2 R2 | Homo sapiens | Hominidae | 150 | 62% | 6E-44 | 51,77% | 238 |
| AAC32312.1 | ubiquitin conjugating enzyme G2 | Homo sapiens | Hominidae | 147 | 71% | 7E-44 | 47,50% | 165 |
| NP_004350.1 | ubiquitin-conjugating enzyme E2 R1 | Homo sapiens | Hominidae | 148 | 63% | 3E-43 | 50,70% | 236 |
| XP_006723015.1 | ubiquitin-conjugating enzyme E2 R1 isoform X2 | Homo sapiens | Hominidae | 146 | 61% | 4E-43 | 51,80% | 179 |
| XP_005259747.1 | ubiquitin-conjugating enzyme E2 R1 isoform X1 | Homo sapiens | Hominidae | 143 | 62% | 4E-41 | 51,06% | 269 |
| NP_872630.1 | ubiquitin-conjugating enzyme E2 G2 isoform 2 | Homo sapiens | Hominidae | 139 | 62% | 6E-41 | 50,71% | 137 |
| NP_003333.1 | ubiquitin-conjugating enzyme E2 G1 | Homo sapiens | Hominidae | 140 | 71% | 7E-41 | 43,75% | 170 |
| NP_650309.1 | ubiquitin conjugating enzyme 87F | Drosophila melanogaster | Drosophilidae | 143 | 74% | 6E-43 | 46,15% | 168 |
| NP_001285334.1 | ubiquitin conjugating enzyme 7, isoform B | Drosophila melanogaster | Drosophilidae | 141 | 72% | 2E-42 | 42,59% | 167 |
| AJS71948.1 | Ubc7p | Saccharomyces cerevisiae, YJM693 | Saccharo-mycetaceae | 132 | 72% | 8E-38 | 44,85% | 165 |
| NP_013735.1 | E2 ubiquitin-conjugating protein UBC7 | Saccharomyces cerevisiae, S288C | Saccharo-mycetaceae | 132 | 72% | 9E-38 | 44,85% | 165 |
| non-ASFV virus (class Megaviricetes) | | | | | | | | |
| accession nr | **accession description** | **organism** | **family** | **max/total score** | **query cover** | **E-value** | **% identity** | **nr. res.** |
| ULY68493.1 | ubiquitin-conjugating enzyme E2 7 | Chlorella virus, XW01 (China, freshwater) | Mimiviridae | 128 | 71% | 2E-34 | 44.65% | 160 |
| QBK86330.1 | ubiquitin-conjugating enzyme E2 | Marseillevirus, LCMAC102 (Norway, hydro-thermal vent) | Marseille-viridae | 108 | 60% | 5E-27 | 41.22% | 148 |
| ARF09360.1 | ubiquitin-conjugating enzyme E2 | Catovirus, CTV1 (South Africa, wastewater) | Mimiviridae | 107 | 69% | 2E-26 | 34.67% | 156 |
| QBK85952.1 | ubiquitin-conjugating enzyme E2 | Marseillevirus, LCMAC101 (Norway, hydro-thermal vent) | Marseille-viridae | 106 | 59% | 7E-26 | 43.41% | 153 |
| YP_003969690.1 | putative ubiquitin-conjugating enzyme E2 | Cafeteria roenbergensis virus, BV-PW1 (USA, seawater) | Mimiviridae | 105 | 49% | 1E-25 | 55.45% | 158 |
| AYV75765.1 | ubiquitin-conjug. enzyme  E2-17 kDa 3 | Terrestrivirus sp., TEV1 (USA, forest soil) | Mimiviridae | 105 | 59% | 2E-25 | 40.16% | 155 |
| QBK87822.1 | ubiquitin-conjugating enzyme E2 | Marseillevirus, LCMAC202 (Norway, hydro-thermal vent) | Marseille-viridae | 103 | 61% | 5E-25 | 40.15% | 148 |
| YP_003969913.1 | putative ubiquitin-conjugating enzyme E2 | Cafeteria roenbergensis virus, BV-PW1* | Mimiviridae | 103 | 63% | 8E-25 | 39.86% | 150 |
| YP_009173309.1 | ubiquitin conjugating enzyme E2 | Chrysochromulina ericina virus, CeV-01B (Norway, seawater) | Phycodna-viridae | 102 | 57% | 2E-24 | 39.02% | 150 |
| ULY68577.1 | ubiquitin-conjugating enzyme E2 2-like isoform X1 | Chlorella virus, XW01* | Mimiviridae | 99 | 46% | 4E-23 | 45.54% | 144 |

**Table S2 -** Curated selection of predicted ubiquitin-conjugating protein sequences from non-ASFV Asfarviridae and Poxviridae virus families, both composing the Pokkesviricetes virus class. Identified ubiquitin-conjugating proteins were found with the NCBI Protein word search of “ubiquitin-conjugating” combined with “poxviridae”, or asfarviridae viruses “abalone asfa-like virus”, “kaumoebavirus”, “faustovirus”, “pacmanvirus”, “tornadovirus”. An additional group of unidentified proteins, marked with an asterisk, was inferred with a TBLASTN search of homologous proteins using Ba71V ASFV-pI215L (NCBI accession AAA65370.1) or identified Asfarviridae and Poxviridae ubiquitin-conjugating proteins (YP_ 009001634.1, SMH63369.1, SIP85775.1) as query sequences, with the search restricted to organisms Poxviridae (taxid 10240, 40069, 2717630), Asfarviridae (taxid 137992, 697905), excluding African Swine Fever virus (10497), Faustovirus (taxid 1477405), Pacmavirus (1932881, 2862371), Abalone asfa-like virus (taxid 2839893), Kaumoebavirus (taxid 1859492), and Alphaentomopoxvirus (taxid 62098, 62099). An additional word search for complete genomes at the NCBI Nucleotide database further indicated the absence of ubiquitin-conjugating proteins in abalone asfa-like virus or kaumoebavirus, and absence of other available faustovirus, pacmanvirus, tornadovirus or alphaentomopoxvirus genomes up to the date of search (July 2024). A total of 22 proteins (13 identified, 9 infered by homology), and 13 distinct protein sequences were retrieved.

| non-ASFV Asfarviridae and Poxviridae (class Pokkesviricetes) | | | | | | |
| --- | --- | --- | --- | --- | --- | --- |
| protein  accession | **name** | **virus, isolate** | **genome accession** | **virus family** | **host** | **loca-tion** |
| YP_ 009001634.1 | ubiquitin-conjugating enzyme E2 | Alphaento-mopoxvirus acuprea, CV6M | NC_023426.1 | Poxviridae | Anomala cuprea (bettle) | Japan |
| AMP44368.1 | hypothetical protein PRJ_Dakar_00417 * | Faustovirus, D3 | KU556803.1 | Asfarviridae | Vermamoeba vermiformis | Senegal, wastewater |
| AMN83985.1 | hypothetical protein D5a_00066 * | Faustovirus, D5a | KU702950.1 | Asfarviridae | Vermamoeba vermiformis | Senegal, wastewater |
| AMN83845.1 | hypothetical protein D5b_00426 * | Faustovirus, D5b | KU702949.1 | Asfarviridae | Vermamoeba vermiformis | Senegal, wastewater |
| AMN84490.1 | hypothetical protein D6_00079 * | Faustovirus, D6 | KU702951.1 | Asfarviridae | Vermamoeba vermiformis | Senegal, wastewater |
| QJX73855.1 | hypothetical protein  F-E9_82 * | Faustovirus, E9 | MT335755.1 | Asfarviridae | Vermamoeba vermiformis | France, wastewater |
| AIB51769.1 | hypothetical protein PRJ_Fausto_00059 * | Faustovirus, E12 | KJ614390.1 | Asfarviridae | Vermamoeba vermiformis | France, wastewater |
| AMN84969.1 | hypothetical protein E23_00066 * | Faustovirus, E23 | KU702952.1 | Asfarviridae | Vermamoeba vermiformis | France, wastewater |
| AMN82999.1 | hypothetical protein E24_00066 * | Faustovirus, E24 | KU702948.1 | Asfarviridae | Vermamoeba vermiformis | France, wastewater |
| SMH63369.1 | Putative Ubiquitin-conjugating enzyme E2 | Faustovirus, LC9 | CZDJ 02000003.1 | Asfarviridae | Vermamoeba vermiformis | France, wastewater |
| QJX71329.1 | ubiquitin-conjugating enzyme | Faustovirus, LCD7 | MN830294.1 | Asfarviridae | Vermamoeba vermiformis | France, wastewater |
| QJX70824.1 | hypothetical protein  F-liban_55 * | Faustovirus, liban | MN534311.1 | Asfarviridae | Vermamoeba vermiformis | Lebanon, seawater |
| QJX71844.1 | ubiquitin-conjugating enzyme E2 | Faustovirus, M6 | MN830295.1 | Asfarviridae | Vermamoeba vermiformis | France, wastewater |
| QBR98972.1 | ubiquitin-conjugating enzyme E2 J1 | Faustovirus, mariensis  PLBH-3D | MK506267.1 | Asfarviridae | Vermamoeba vermiformis | Brasil, wastewater |
| QJX72331.1 | ubiquitin-conjugating enzyme E2 | Faustovirus, S17 | MN830296.1 | Asfarviridae | Vermamoeba vermiformis | Algeria, wastewater |
| SME64724.1 | Putative Ubiquitin-conjugating enzyme E2 | Faustovirus, ST1 | LT839607.1 | Asfarviridae | Vermamoeba vermiformis | France, wastewater |
| QKE50186.1 | ubiquitin-conjugating enzyme E2 | Faustovirus, VV10 | MN956669.1 | Asfarviridae | Vermamoeba vermiformis | Algeria, freshwater |
| QJX72841.1 | ubiquitin-conjugating enzyme E2 | Faustovirus, VV57 | MN830297.1 | Asfarviridae | Vermamoeba vermiformis | Algeria, freshwater |
| QJX73347.1 | ubiquitin-conjugating enzyme E2 | Faustovirus, VV63 | MN830298.1 | Asfarviridae | Vermamoeba vermiformis | Algeria, wastewater |
| SIP85775.1 | Ubiquitin-conjugating enzyme E2 | Pacmanvirus, A23 | LT706986.1 | (Asfarviridae) | Acanthamoeba castellanii | Algeria, wastewater |
| QYB17424.1 | E2 ubiquitin-conjugating enzyme | Pacmanvirus, S19 | MZ440852.1 | (Asfarviridae) | Acanthamoeba castellanii | Algeria, wastewater |
| BFG87710.1 | E2 ubiquitin-conjugating enzyme | Tornadovirus japonicus, D1 | LC801470.1 | (Asfarviridae) | Acanthamoeba castellanii | Japan, freshwater |

**Table S3 -** Curated selection of ASFV-pI215L protein sequences of different ASFV strains and isolates. A NCBI Nucleotide database word search for “ASFV genome” or “african swine fever genome” delivered 222 results at the time of search (June 2023) after exclusion of redundant accessions, partial genome or genes, non-natural viruses, among others. The NCBI Protein accession of ASFV-pI215L encoded by each genome was collected after searching for protein annotations available in the genome accession. In cases where genome annotation was absent, the ASFV-pI215L sequence was inferred through a NCBI TBLASTN alignment of the genome with Ba71V ASFV-pI215L (NCBI accession AAA65370.1).

| ASFV genome | genome accession | genotype | pI215L accession |
| --- | --- | --- | --- |
| K49, Congo 1949 | MZ202520.1 | I | QZK26842.1 |
| L60, Portugal 1960 | KM262844.1 | I | AIY22332.1 |
| NH/P68 (or NHV), Portugal 1968 | KM262845.1 | I | AIY22490.1 |
| Ba71, Spain 1971 | KP055815.1 | I | AKO62822.1 |
| Ba71V (Vero-adapted), Spain 1971 | U18466.2 | I | AAA65370.1 |
| E75, Spain 1975 | FN557520.1 | I | CBH29241.1 |
| DR/1979, Dominican Republic 1979 | ON185726.1 | I | USZ79734.1 |
| Dr-1980, Dominican Republic 1980 | ON185726.2 | I | USZ79734.1 |
| LIV_5_40, Zambia 1983 | MN318203.3 | I | QRW44281.1 |
| Liv13/33 (OmLF2), Zambia 1983 | MN913970.1 | I | QID21336.1 |
| OURT 88/3, Portugal 1988 | AM712240.1 | I | CAN10490.1 |
| Benin 97/1, Benin 1997 | AM712239.1 | I | CAN10240.1 |
| Arm/07/CBM/c4, Armenia 2007 | LR881473.1 | I | CAD5338279.1 |
| LO2018 major, Italy 2018 | MW647171.1 | I | UFQ11373.1 |
| LO2018 minor, Italy 2018 | MW647172.1 | I | UFQ11602.1 |
| ASF/IND/20/CAD/543, India 2020 | OK236383.1 | I | inferred from homology |
| Pig/HeN/ZZ-P1/2021, China 2021 | MZ945536.1 | I | UEN73150.1 |
| Pig/SD/DY-I/2021, China 2021 | MZ945537.1 | I | UEN73308.1 |
| Russia 2022 | OM249788.1 | I | WAS30585.1 |
| 56/Ca/1978, Italy (Sardinia) 1978 | MN270969.1 | I | QIM06850.1 |
| Ca1978_2, Italy (Sardinia) 1978 | MW723480.1 | I | UCX48426.1 |
| 57/Ca/1979, Italy (Sardinia) 1979 | MN270970.1 | I | QIM07085.1 |
| Nu1979, Italy (Sardinia) 1979 | MW723481.1 | I | UCX48650.1 |
| 139/Nu/1981, Italy (Sardinia) 1981 | MN270971.1 | I | QIM07320.1 |
| 140/Or/1985, Italy (Sardinia) 1981 | MN270972.1 | I | QIM07555.1 |
| SS_1981, Italy (Sardinia) 1981 | MW788409.1 | I | UCX58351.1 |
| Or_1984, Italy (Sardinia) 1984 | MW800838.1 | I | UCX58810.1 |
| 85/Ca/1985, Italy (Sardinia) 1985 | MN270973.1 | I | QIM07788.1 |
| Nu1986, Italy (Sardinia) 1986 | MW723482.1 | I | UCX48879.1 |
| 141/Nu/1990, Italy (Sardinia) 1990 | MN270974.1 | I | QIM08021.1 |
| Nu1990_1, Italy (Sardinia) 1990 | MW723483.1 | I | UCX49107.1 |
| Nu1991_2, Italy (Sardinia) 1991 | MW723484.1 | I | UCX49338.1 |
| Nu1991_3, Italy (Sardinia) 1991 | MW723485.1 | I | UCX49569.1 |
| Nu1991_7, Italy (Sardinia) 1991 | MW723486.1 | I | UCX49800.1 |
| Nu1993_2, Italy (Sardinia) 1993 | MW723488.1 | I | UCX50261.1 |
| Or1993_1, Italy (Sardinia) 1993 | MW723487.1 | I | UCX50031.1 |
| 142/Nu/1995, Italy (Sardinia) 1995 | MN270975.1 | I | QIM08256.1 |
| Nu1995_2, Italy (Sardinia) 1995 | MW723489.1 | I | UCX50491.1 |
| Nu1995_3, Italy (Sardinia) 1995 | MW723490.1 | I | UCX50720.1 |
| Nu1995_4, Italy (Sardinia) 1995 | MW723491.1 | I | UCX50950.1 |
| 60/Nu/1997, Italy (Sardinia) 1997 | MN270976.1 | I | QIM08489.1 |
| 24225_2002, Italy (Sardinia) 2002 | MW788411.1 | I | inferred from homology |
| 26/Ss/2004, Italy (Sardinia) 2004 | MN270977.1 | I | QIM08722.1 |
| 44076, Italy (Sardinia) 2004 | MW723500.1 | I | UCX53030.1 |
| 74377, Italy (Sardinia) 2004 | MW723496.1 | I | UCX52105.1 |
| 22649, Italy (Sardinia) 2005 | MW723497.1 | I | UCX52337.1 |
| 31479_2005, Italy (Sardinia) 2005 | MW788407.1 | I | UCX57896.1 |
| 72398 WB, Italy (Sardinia) 2005 | MW723495.1 | I | UCX51874.1 |
| 72407/Ss/2005, Italy (Sardinia) 2005 | MN270978.1 | I | QIM08955.1 |
| 72912 WB, Italy (Sardinia) 2007 | MW723498.1 | I | UCX52568.1 |
| 1537 WB, Italy (Sardinia) 2008 | MW788405.1 | I | UCX57440.1 |
| 22137, Italy (Sardinia) 2008 | MW723499.1 | I | UCX52799.1 |
| 22943_2008, Italy (Sardinia) 2008 | MW788406.1 | I | UCX57669.1 |
| 23221, Italy (Sardinia) 2008 | MW723494.1 | I | UCX51643.1 |
| 25185_2008, Italy (Sardinia) 2008 | MW788410.1 | I | UCX58581.1 |
| 46830, Italy (Sardinia) 2008 | MW723493.1 | I | UCX51412.1 |
| 47/Ss/2008, Italy (Sardinia) 2008 | KX354450.1 | I | AOO54513.1 |
| 4996 WB, Italy (Sardinia) 2008 | MW723492.1 | I | UCX51181.1 |
| 26544/OG10, Italy (Sardinia) 2010 | KM102979.1 | I | AJZ77128.1 |
| 31208, Italy (Sardinia) 2011 | MW736612.1 | I | UCX56729.1 |
| 2019 WB, Italy (Sardinia) 2012 | MW736598.1 | I | UCX53492.1 |
| 63525 WB, Italy (Sardinia) 2012 | MW736603.1 | I | UCX54648.1 |
| 97/Ot/2012, Italy (Sardinia) 2012 | MN270979.1 | I | QIM09188.1 |
| 113049 WB, Italy (Sardinia) 2013 | MW736608.1 | I | UCX55805.1 |
| 30322, Italy (Sardinia) 2013 | MW736600.1 | I | UCX53954.1 |
| 32516, Italy (Sardinia) 2013 | MW736607.1 | I | UCX55573.1 |
| ASFV genome | **genome accession** | **genotype** | **pI215L accession** |
| 47039, Italy (Sardinia) 2013 | MW736597.1 | I | UCX53261.1 |
| 49179 WB, Italy (Sardinia) 2013 | MW736601.1 | I | UCX54185.1 |
| 98039, Italy (Sardinia) 2013 | MW736599.1 | I | UCX53723.1 |
| 22653/Ca/2014, Italy (Sardinia) 2014 | MN270980.1 | I | QIM09421.1 |
| 35479_2014, Italy (Sardinia) 2014 | MW788408.1 | I | UCX58122.1 |
| 51268, Italy (Sardinia) 2014 | MW736605.1 | I | UCX55110.1 |
| 15998, Italy (Sardinia) 2015 | MW736604.1 | I | UCX54879.1 |
| 28928, Italy (Sardinia) 2015 | MW736610.1 | I | UCX56266.1 |
| 33262WB, Italy (Sardinia) 2015 | ON260841.1 | I | WHO20034.1 |
| 33747 WB, Italy (Sardinia) 2015 | MW736613.1 | I | UCX56960.1 |
| 6396 WB, Italy (Sardinia) 2015 | MW736609.1 | I | UCX56036.1 |
| 28784WB, Italy (Sardinia) 2016 | ON260840.1 | I | WHO19818.1 |
| 53706, Italy (Sardinia) 2016 | MW736602.1 | I | UCX54416.1 |
| 34403, Italy (Sardinia) 2017 | MW736606.1 | I | UCX55341.1 |
| 103917/18, Italy (Sardinia) 2018 | MT932578.1 | I | QPL11885.1 |
| 55234/18, Italy (Sardinia) 2018 | MT932579.1 | I | QPL12102.1 |
| 56140, Italy (Sardinia) 2018 | MW736611.1 | I | UCX56497.1 |
| 7212WB, Italy (Sardinia) 2019 | ON260838.2 | I | WAF58251.1 |
| 7303WB, Italy (Sardinia) 2019 | ON260839.2 | I | WAF58023.1 |
| Arm/07/CBM/c2, Armenia 2007 | LR812933.1 | II | inferred from homology |
| Georgia 2007/1, Georgia 2007 | FR682468.2 | II | CAD2068503.1 |
| Georgia 2008/1, Georgia 2008 | MH910495.1 | II | AZP54049.1 |
| Georgia 2008/2, Georgia 2008 | MH910496.1 | II | AZP54228.1 |
| Kashino 04/13, Russia 2013 | KJ747406.1 | II | inferred from homology |
| ASFV/LT14/1490, Lithuania 2014 | MK628478.1 | II | QEY87947.1 |
| Estonia 2014, Estonia 2014 | LS478113.1 | II | SPS73564.1 |
| Odintsovo_02/14, Russia 2014 | KP843857.1 | II | inferred from homology |
| ASFV/POL/2015/Podlaskie, Poland 2015 | MH681419.1 | II | inferred from homology |
| ASFV/Kyiv/2016/131, Ukraine 2016 | MN194591.1 | II | QED21757.1 |
| Pol16_20186_o7, Poland 2016 | MG939583.1 | II | AXZ95912.1 |
| Pol16_20538_o9, Poland 2016 | MG939584.1 | II | inferred |
| Pol16_20540_o10, Poland 2016 | MG939585.1 | II | inferred |
| Pol16_29413_o23, Poland 2016 | MG939586.1 | II | inferred |
| ASFV/Kaliningrad_17/WB-13869, Russia 2017 | OM799941.1 | II | UVI02204.1 |
| CzechRepublic 2017/1, Czech Republic 2017 | LR722600.1 | II | VVW94288.1 |
| Krasnodar 07/17, Russia 2017 | MH894399.1 | II | QCE30325.1 |
| Moldova 2017/1, Moldova 2017 | LR722599.1 | II | VVW94283.1 |
| Pol17_03029_C201, Poland 2017 | MG939587.1 | II | inferred from homology |
| Pol17_04461_C210, Poland 2017 | MG939588.1 | II | AXZ96195.1 |
| Pol17_05838_C220, Poland 2017 | MG939589.1 | II | inferred from homology |
| Pol17_31177_O81, Poland 2017 | MT847622.1 | II | QOW03063.1 |
| Pol17_55892_C754, Poland 2017 | MT847620.1 | II | QOW02688.1 |
| Tanzania/Rukwa/2017/1, Tanzania 2017 | LR813622.1 | II | CAD0059592.1 |
| ASFV_HU_2018, Hungary 2018 | MN715134.1 | II | QGV56898.1 |
| ASFV/Kaliningrad_18/WB-12516, Russia 2018 | OM966720.1 | II | UVH35842.1 |
| ASFV/Kaliningrad_18/WB-12523, Russia 2018 | OM966714.1 | II | UVH34749.1 |
| ASFV/Kaliningrad_18/WB-12524, Russia 2018 | OM966715.1 | II | UVH34931.1 |
| ASFV/Kaliningrad_18/WB-9734, Russia 2018 | OM966721.1 | II | UVH36024.1 |
| ASFV/Kaliningrad_18/WB-9735, Russia 2018 | OM966716.1 | II | UVH35113.1 |
| ASFV/Kaliningrad_18/WB-9763, Russia 2018 | OM966717.1 | II | UVH35295.1 |
| ASFV/Kaliningrad_18/WB-9766, Russia 2018 | OM966718.1 | II | UVH35477.1 |
| ASFV-SY18, China 2018 | MH766894.2 | II | inferred from homology |
| ASFV-wbBS01, China 2018 | MK645909.1 | II | QDL88172.1 |
| Belgium 2018/1, Belgium 2018 | LR536725.1 | II | VFV48075.1 |
| Belgium/Etalle/wb/2018, Belgium 2018 | MK543947.1 | II | QED90584.1 |
| China/2018/AnhuiXCGQ, China 2018 | MK128995.1 | II | AYW34112.1 |
| DB/LN/2018, China 2018 | MK333181.1 | II | QBH90814.1 |
| GZ201801, China 2018 | MT496893.1 | II | QLF78669.1 |
| GZ201801_2, China 2018 | ON263123.1 | II | UUW33327.1 |
| LYG18, China 2018 | OM105586.1 | II | UZT04531.1 |
| Pig/HLJ/2018, China 2018 | MK333180.1 | II | QBH90629.1 |
| Pol18_28298_O111, Poland 2018 | MT847621.1 | II | QOW02877.1 |
| ASFV/Amur 19/WB-6905, Russia 2019 | MW306190.1 | II | QUQ60246.1 |
| ASFV_Hanoi_2019, Vietnam 2019 | MT166692.1 | II | QOY24245.1 |
| ASFV/Kabardino-Balkaria 19/WB-964, Russia 2019 | MT459800.1 | II | QPB67667.1 |
| ASFV/Kaliningrad_19/WB-10168, Russia 2019 | OM966719.1 | II | UVH35660.1 |
| ASFV/Korea/pig/PaJu1/2019, South Korea 2019 | MT748042.1 | II | inferred from homology |
| ASFV_NgheAn_2019, Vietnam 2019 | MT180393.1 | II | QOY24446.1 |
| ASFV/pig/China/CAS19-01/2019, China 2019 | MN172368.1 | II | QGJ83503.1 |
| ASFV/Primorsky 19/WB-6723, Russia 2019 | MW306191.1 | II | QUQ60426.1 |
| ASFV/Timor-Leste/2019/1, Timor-Leste 2019 | MW396979.1 | II | QTE18843.1 |
| ASFV/Ulyanovsk 19/WB-5699, Russia 2019 | MW306192.1 | II | QUQ60606.1 |
| ASFV-wbShX01, China 2019 | MW033528.1 | II | UMA73245.1 |
| CADC_HN09, China 2019 | MZ614662.1 | II | UFD97909.1 |
| China/GD/2019, China 2019 | MW361944.1 | II | inferred from homology |
| CN/2019/InnerMongolia-AES01, China 2019 | MK940252.1 | II | QIA61555.1 |
| Korea/pig/Yeoncheon1/2019, Korea 2019 | MW049116.1 | II | UID85854.1 |
| Korea/YC1/2019, South Korea 2019 | ON075797.1 | II | URS64878.1 |
| ASFV genome | **genome accession** | **genotype** | **pI215L accession** |
| MAL/19/Karonga, Malawi 2019 | MW856068.1 | II | QXP50100.1 |
| Pol19_53050_C1959/19, Poland 2019 | MT847623.1 | II | QOW03249.1 |
| VN/HY-ASFV1(2019), Vietnam 2019 | MT872723.1 | II | UDM55670.1 |
| VN/QP-ASFV1(2019), Vietnam 2019 | MT882025.1 | II | UDP03423.1 |
| Wuhan 2019-1, China 2019 | MN393476.1 | II | QIE06930.1 |
| Wuhan 2019-2, China 2019 | MN393477.1 | II | QIE07093.1 |
| 2020ASP01832, Germany 2020 | OX376256.1 | II | CAI4201462.1 |
| 2021ASP01917, Germany 2020 | OX376260.1 | II | CAI4201459.1 |
| 2020ASP02805, Germany 2020 | OX376250.1 | II | CAI4200669.1 |
| 2020ASP02894, Germany 2020 | OX376258.1 | II | CAI4201467.1 |
| ABTCVSCK_ASF001, India 2020 | OM481275.1 | II | UPH95601.1 |
| ASFV/Zabaykali/WB-5314/2020, Russia 2020 | MZ325862.1 | II | inferred from homology |
| Germany 2020/1, Germany 2020 | LR899193.1 | II | CAD7112652.1 |
| HB31A, China 2020 | ON380540.1 | II | UYE97716.1 |
| HB03A, China 2020 | ON380539.1 | II | UYE97536.1 |
| HuB20, China 2020 | MW521382.1 | II | QTP96449.1 |
| IND/AS/SD-02/2020, India 2020 | OL692743.1 | II | UNZ12275.1 |
| IND/AR/SD-61/2020, India 2020 | OL692744.1 | II | UNZ12494.1 |
| Korea/HC224/2020, South Korea 2020 | OP628183.1 | II | UZS00769.1 |
| Pig/Heilongjiang/HRB1/2020, China 2020 | MW656282.1 | II | QST88225.1 |
| SY-1, China 2020 | OM161110.1 | II | UYC33215.1 |
| Vietnam/Pig/RG-1/01, 2020 | OL322096.1 | II | inferred from homology |
| Vietnam/Pig/RG-2/01, 2020 | OL322097.1 | II | inferred from homology |
| Vietnam/Pig/RG-3/01, 2020 | OL322098.1 | II | inferred from homology |
| Vietnam/Pig/RG-4/01, 2020 | OL322099.1 | II | inferred from homology |
| Vietnam/Pig/RG-5/01, 2020 | OL322100.1 | II | inferred from homology |
| Vietnam/Pig/RG-6/01, 2020 | OL322101.1 | II | inferred from homology |
| Vietnam/Pig/RG-7/01, 2020 | OL322102.1 | II | inferred from homology |
| VNUA-ASFV-05L1/HaNam/VN/2020, Vietnam 2020 | MW465755.1 | II | QSG73743.1 |
| 2021ASP00484, Germany 2021 | OX376254.1 | II | CAI4200628.1 |
| 2021ASP00703, Germany 2021 | OX376252.1 | II | CAI4200681.1 |
| 2021ASP00902, Germany 2021 | OX376255.1 | II | CAI4200641.1 |
| 2021ASP00921, Germany 2021 | OX376262.1 | II | CAI4201465.1 |
| 2021ASP01919, Germany 2021 | OX376251.1 | II | CAI4200733.1 |
| 2021ASP01957, Germany 2021 | OX376253.1 | II | CAI4200668.1 |
| 2020ASP02103, Germany 2021 | OX376261.1 | II | CAI4201457.1 |
| 2021ASP02148, Germany 2021 | OX376257.1 | II | CAI4201424.1 |
| 2021ASP02207, Germany 2021 | OX376263.1 | II | CAI4201450.1 |
| 2021ASP02665, Germany 2021 | OX376259.1 | II | CAI4201437.1 |
| 2021ASP03144, Germany 2021 | OX376268.1 | II | CAI4209972.1 |
| 2021ASP03251, Germany 2021 | OX376267.1 | II | CAI4209964.1 |
| 2021ASP03380, Germany 2021 | OX376264.1 | II | CAI4209926.1 |
| 2021ASP03384, Germany 2021 | OX376265.1 | II | CAI4209951.1 |
| 2021ASP03643, Germany 2021 | OX376266.1 | II | CAI4209937.1 |
| 2021ASP03658, Germany 2021 | OX376271.1 | II | CAI4209999.1 |
| 2021ASP03711, Germany 2021 | OX376273.1 | II | CAI4210073.1 |
| 2021ASP03740, Germany 2021 | OX376272.1 | II | CAI4210056.1 |
| (serotype 8 genotype 2), Vietnam 2021 | ON402789.1 | II | inferred from homology |
| A4, Philippines 2021 | ON963982.1 | II | UUH61811.1 |
| ABTCVSCK_ASF007, India 2021 | OM481276.1 | II | UPH95796.1 |
| AQS-P-201202 DNA, Japan 2021 | LC659089.1 | II | BDC47376.1 |
| AQS-P-20901-1 DNA, Japan 2021 | LC659088.1 | II | BDC47193.1 |
| AQS-C-1-21 DNA, Japan 2021 | LC659086.1 | II | BDC46827.1 |
| AQS-C-1-22 DNA, Japan 2021 | LC659087.1 | II | BDC47010.1 |
| SY-2, China 2021 | OP612151.1 | II | UZP65547.1 |
| 2802/AL/2022 Italy, Italy 2022 | ON108571.3 | II | UPT51815.1 |
| SPEC_57, South Africa 1985 | MN394630.3 | III | QRW43544.1 |
| Warmbaths, South Africa 1987 | AY261365.1 | III | inferred from homology |
| Warthog/Namibia/Wart80/1980 | AY261366.1 | IV | inferred from homology |
| RSA_W1_1999, South Africa 1999 | MN641876.1 | IV | inferred from homology |
| Tengani 62, Malawi 1962 | AY261364.1 | V | inferred from homology |
| Mkuzi 1979, South Africa 1979 | AY261362.1 | VII | inferred from homology |
| Malawi Lil-20/1 (1983), Malawi 1983 | AY261361.1 | VIII | inferred from homology |
| R8, Uganda 2015 | MH025916.1 | IX | AXB49369.1 |
| R7, Uganda 2015 | MH025917.1 | IX | AXB49543.1 |
| R25, Uganda 2015 | MH025918.1 | IX | AXB49715.1 |
| N10, Uganda 2015 | MH025919.1 | IX | AXB49886.1 |
| R35, Uganda 2015 | MH025920.1 | IX | AXB50059.1 |
| Ken06.Bus, Kenya 2006 | KM111295.1 | IX | AJL34317.1 |
| Ken05/Tk1, Kenya 2005 | KM111294.1 | X | AJL34153.1 |
| Ken.rie1, Kenya 2020 | LR899131.1 | X | CAD7112356.1 |
| Kenya 1950, Kenya 1950 | AY261360.1 | X | inferred from homology |
| BUR/18/Rutana, Burundi 2018 | MW856067.1 | X | QXP49923.1 |
| Uvira B53, Congo 2019 | MT956648.1 | X | QRY19166.1 |
| Pretoriuskop/96/4, South Africa 1996 | AY261363.1 | XX | inferred from homology |
| Zaire, Zaire 1977 | MN630494.1 | XX | inferred from homology |
| RSA_2_2004, South Africa 2004 | MN641877.1 | XX | inferred from homology |
| RSA_2_2008, South Africa 2008 | MN336500.3 | XXII | inferred from homology |

**Table S4 -** Detailed information of all 32 expressed recombinant ASFV-pI215L proteins intended for ubiquitination assays. After inserting the mutation, ASFV-pI215L sequences were codon optimized for E. *coli* expression. The NCBI database was used to garner the DNA sequence of the ORF I215L encoded by Ba71V ASFV (Gene ID: 22220370) or NH/P68 (NHV) ASFV (Gene ID: 41901511). pET-24a+ plasmids expressing single-point and multi-point mutants were synthesized by Genscript, and epitope mutants were synthesized by Azenta.

| single-point mutants | | | | | |
| --- | --- | --- | --- | --- | --- |
| protein name | **pI215L sequence** | **res. position of mutation** | **native sequence** | **altered sequence** | **nr altered residues** |
| WT | Ba71V | no alterations made to ORF | | | 0 |
| R4S | Ba71V | 4 | R | S | 1 |
| R4E | Ba71V | 4 | R | E | 1 |
| R11E | Ba71V | 11 | R | E | 1 |
| R11A | Ba71V | 11 | R | A | 1 |
| S87D | Ba71V | 87 | S | D | 1 |
| S87R | Ba71V | 87 | S | R | 1 |
| E120R | Ba71V | 120 | E | R | 1 |
| E120S | Ba71V | 120 | E | S | 1 |
| S125D | Ba71V | 125 | S | D | 1 |
| S125R | Ba71V | 125 | S | R | 1 |
| D130R | Ba71V | 130 | D | R | 1 |
| D130S | Ba71V | 130 | D | S | 1 |
| multi-point mutants | | | | | |
| protein name | **pI215L sequence** | **res. position of mutation** | **native sequence** | **altered sequence** | **nr altered residues** |
| 11A | Ba71V | 11-15 | RHLIE | ARILD | 5 |
| 11B | Ba71V | 11-15 | RHLIE | AKVVD | 5 |
| 11C | Ba71V | 11-15 | RHLIE | AQTTQ | 5 |
| 130A | Ba71V | 130-134 | DAAKS | SVVRT | 5 |
| 130B | Ba71V | 130-134 | DAAKS | SIIHN | 5 |
| 130C | Ba71V | 130-134 | DAAKS | SSSQA | 5 |
| 11A+130A | Ba71V | 11-15  130-134 | RHLIE  DAAKS | ARILD  SVVRT | 10 |
| epitope mutants | | | | | |
| protein name | **pI215L sequence** | **res. position of mutation** | **native sequence** | **altered sequence** | **nr altered residues** |
| WT Ba71V | Ba71V | no alterations made to ORF | | | 0 |
| WT NHV | NH/P68 | no alterations made to ORF | | | 0 |
| E | NH/P68 | 61-69 | PYAPPKLTF | PEDPPKLTF | 2 |
| E+11A | NH/P68 | 61-69  11-15 | PYAPPKLTF  RHLIE | PEDPPKLTF  ARILD | 7 |
| E+11B | NH/P68 | 61-69  11-15 | PYAPPKLTF  RHLIE | PEDPPKLTF  AKVVD | 7 |
| E+11C | NH/P68 | 61-69  11-15 | PYAPPKLTF  RHLIE | PEDPPKLTF  AQTTQ | 7 |
| E+130A | NH/P68 | 61-69  130-134 | PYAPPKLTF  DAAKS | PEDPPKLTF  SSSQA | 7 |
| Q | NH/P68 | 61-69 | PYAPPKLTF | PNSPPQTLQ | 6 |
| Q+11A | NH/P68 | 61-69  11-15 | PYAPPKLTF  RHLIE | PNSPPQTLQ  ARILD | 11 |
| Q+11B | NH/P68 | 61-69  11-15 | PYAPPKLTF  RHLIE | PNSPPQTLQ  AKVVD | 11 |
| Q+11C | NH/P68 | 61-69  11-15 | PYAPPKLTF  RHLIE | PNSPPQTLQ  AQTTQ | 11 |
| Q+130A | NH/P68 | 61-69  130-134 | PYAPPKLTF  DAAKS | PNSPPQTLQ  SSSQA | 11 |

**Table S5 -** Detailed information of the peptide libraries designed for the ELISA assays, synthesized by Pepscan. The Biotin-tag library is composed of 39 peptides of 25-residue ASFV-pI215L segments, with a 5-residue offset and 20-residue overlap, conjugated with an N-terminal Biotin-Ahx- tag. The PS-tag library comprises 43 peptides of 9-residue ASFV-pI215L segments, with a 5-residue offset and a 4-residue overlap, synthesized with a 12 residue C-terminal polystyrene-binding peptide and a 4-glycine spacer (-GGGGRAFIASRRIRRP). The Ba71V ASFV-pI215L sequence was retrieved from Uniprot (P27949).

| biotin-tag library | | |  | PS-tag library | | |
| --- | --- | --- | --- | --- | --- | --- |
| peptide ID | **pI215L residue position** | **sequence** |  | **peptide ID** | **pI215L residue position** | **sequence** |
| biot-1 | 1-25 | MVSRFLIAEYRHLIENPSENFKISV |  | **ps-1** | 1-9 | MVSRFLIAE |
| biot-2 | 6-30 | LIAEYRHLIENPSENFKISVNENNI |  | **ps-2** | 6-14 | LIAEYRHLI |
| biot-3 | 11-35 | RHLIENPSENFKISVNENNITEWDV |  | **ps-3** | 11-19 | RHLIENPSE |
| biot-4 | 16-40 | NPSENFKISVNENNITEWDVILRGP |  | **ps-4** | 16-24 | NPSENFKIS |
| biot-5 | 21-45 | FKISVNENNITEWDVILRGPPDTLY |  | **ps-5** | 21-29 | FKISVNENN |
| biot-6 | 26-50 | NENNITEWDVILRGPPDTLYEGGLF |  | **ps-6** | 26-34 | NENNITEWD |
| biot-7 | 31-55 | TEWDVILRGPPDTLYEGGLFKAKVA |  | **ps-7** | 31-39 | TEWDVILRG |
| biot-8 | 36-60 | ILRGPPDTLYEGGLFKAKVAFPPEY |  | **ps-8** | 36-44 | ILRGPPDTL |
| biot-9 | 41-65 | PDTLYEGGLFKAKVAFPPEYPYAPP |  | **ps-9** | 41-49 | PDTLYEGGL |
| biot-10 | 46-70 | EGGLFKAKVAFPPEYPYAPPKLTFT |  | **ps-10** | 46-54 | EGGLFKAKV |
| biot-11 | 51-75 | KAKVAFPPEYPYAPPKLTFTSEMWH |  | **ps-11** | 51-59 | KAKVAFPPE |
| biot-12 | 56-80 | FPPEYPYAPPKLTFTSEMWHPNIYP |  | **ps-12** | 56-64 | FPPEYPYAP |
| biot-13 | 61-85 | PYAPPKLTFTSEMWHPNIYPDGRLC |  | **ps-13** | 61-69 | PYAPPKLTF |
| biot-14 | 66-90 | KLTFTSEMWHPNIYPDGRLCISILH |  | **ps-14** | 66-74 | KLTFTSEMW |
| biot-15 | 71-95 | SEMWHPNIYPDGRLCISILHGDNAE |  | **ps-15** | 71-79 | SEMWHPNIY |
| biot-16 | 76-100 | PNIYPDGRLCISILHGDNAEEQGMT |  | **ps-16** | 76-84 | PNIYPDGRL |
| biot-17 | 81-105 | DGRLCISILHGDNAEEQGMTWSPAQ |  | **ps-17** | 81-89 | DGRLCISIL |
| biot-18 | 86-110 | ISILHGDNAEEQGMTWSPAQKIDTI |  | **ps-18** | 86-94 | ISILHGDNA |
| biot-19 | 91-115 | GDNAEEQGMTWSPAQKIDTILLSVI |  | **ps-19** | 91-99 | GDNAEEQGM |
| biot-20 | 96-120 | EQGMTWSPAQKIDTILLSVISLLNE |  | **ps-20** | 96-104 | EQGMTWSPA |
| biot-21 | 101-125 | WSPAQKIDTILLSVISLLNEPNPDS |  | **ps-21** | 101-109 | WSPAQKIDT |
| biot-22 | 106-130 | KIDTILLSVISLLNEPNPDSPANVD |  | **ps-22** | 106-114 | KIDTILLSV |
| biot-23 | 111-135 | LLSVISLLNEPNPDSPANVDAAKSY |  | **ps-23** | 111-119 | LLSVISLLN |
| biot-24 | 116-140 | SLLNEPNPDSPANVDAAKSYRKYVY |  | **ps-24** | 116-124 | SLLNEPNPD |
| biot-25 | 121-145 | PNPDSPANVDAAKSYRKYVYKEDLE |  | **ps-25** | 121-129 | PNPDSPANV |
| biot-26 | 126-150 | PANVDAAKSYRKYVYKEDLESYPME |  | **ps-26** | 126-134 | PANVDAAKS |
| biot-27 | 131-155 | AAKSYRKYVYKEDLESYPMEVKKTV |  | **ps-27** | 131-139 | AAKSYRKYV |
| biot-28 | 136-160 | RKYVYKEDLESYPMEVKKTVKKSLD |  | **ps-28** | 136-144 | RKYVYKEDL |
| biot-29 | 141-165 | KEDLESYPMEVKKTVKKSLDECSPE |  | **ps-29** | 141-149 | KEDLESYPM |
| biot-30 | 146-170 | SYPMEVKKTVKKSLDECSPEDIEYF |  | **ps-30** | 146-154 | SYPMEVKKT |
| biot-31 | 151-175 | VKKTVKKSLDECSPEDIEYFKNAAS |  | **ps-31** | 151-159 | VKKTVKKSL |
| biot-32 | 156-180 | KKSLDECSPEDIEYFKNAASNVPPI |  | **ps-32** | 156-164 | KKSLDECSP |
| biot-33 | 161-185 | ECSPEDIEYFKNAASNVPPIPSDAY |  | **ps-33** | 161-169 | ECSPEDIEY |
| biot-34 | 166-190 | DIEYFKNAASNVPPIPSDAYEDECE |  | **ps-34** | 166-174 | DIEYFKNAA |
| biot-35 | 171-195 | KNAASNVPPIPSDAYEDECEEMEDD |  | **ps-35** | 171-179 | KNAASNVPP |
| biot-36 | 176-200 | NVPPIPSDAYEDECEEMEDDTYILT |  | **ps-36** | 176-184 | NVPPIPSDA |
| biot-37 | 181-205 | PSDAYEDECEEMEDDTYILTYDDDE |  | **ps-37** | 181-189 | PSDAYEDEC |
| biot-38 | 186-210 | EDECEEMEDDTYILTYDDDEEEEDE |  | **ps-38** | 186-194 | EDECEEMED |
| biot-39 | 191-215 | EMEDDTYILTYDDDEEEEDEEMDDE |  | **ps-39** | 191-199 | EMEDDTYIL |
|  |  |  |  | **ps-40** | 196-204 | TYILTYDDD |
|  |  |  |  | **ps-41** | 201-209 | YDDDEEEED |
|  |  |  |  | **ps-42** | 206-214 | EEEDEEMDD |
|  |  |  |  | **ps-43** | 207-215 | EEDEEMDDE |

**Table S6 -** Detailed information of the PS-tag peptide library intended for ELISA analysis of WT vs mutant sequences of the identified ASFV-pI215L immunogenic region. The wildtype sequence corresponds to peptide ps-13, of ASFV-pI215L residue position 61-69. Peptides described below constitute sequence variations of the 9-residue segment (sequence PYAPPKLTF) with alteration of 2 to 6 residues, synthesized with a C-terminal polystyrene-binding peptide and glycine spacer (Pepscan). The sequence of peptides E and Q selected for design and full-length production of epitope mutants is marked with an asterisk (Table S4).

| peptide ID | nr res. alteration | sequence |  | peptide ID | nr res. alteration | sequence |
| --- | --- | --- | --- | --- | --- | --- |
| A | 2 | PFVPPKLTF |  | **M** | 6 | PQTPPQTVQ |
| B | 2 | PAYPPKLTF |  | **N** | 6 | PRHPPRHKR |
| C | 2 | PQTPPKLTF |  | **O** | 6 | PEDPPEDEE |
| D | 2 | PRHPPKLTF |  | **P** | 6 | PVFPPHYQA |
| E* | 2 | PEDPPKLTF |  | **Q*** | 6 | PNSPPQTLQ |
| F | 4 | PYAPPRISY |  | **R** | 6 | PRRPPEKKK |
| G | 4 | PYAPPHYQA |  | **S** | 6 | PDRPPKDSF |
| H | 4 | PYAPPQTVQ |  | **T** | 4 | PYAPPHIVH |
| I | 4 | PYAPPRHKR |  | **U** | 4 | PYAPPRVLY |
| J | 4 | PYAPPEDEE |  | **V** | 6 | PNSPPESLN |
| K | 6 | PFVPPRISY |  | **W** | 6 | PHSPPEHDK |
| L | 6 | PAYPPHYQA |  | **X** | 6 | PFIPPEKIH |

**Table S7 -** Detailed information of the 94 domestic pig serum samples used for the ELISA assays, provided by INIA-CISA. Anti-p72 antibody detection with INgezim® PPA Compac ELISA kit (Ingenasa). Sera a1 to a82 were collected at different DPI from 12 pigs infected with a genotype II ASFV, used in all ELISA assays (sera were selected according to DPI for the PS-tag screening and ELISA analysis of wildtype vs mutant peptides). Additional sera b1 to b12, each collected from a different pig, were used for the ELISA analysis with individual sera. DPI = days post infection. n.i = not included in sera pools.

| sera ID | pig ID | ASFV strain | virulence | diagno-sis | route | DPI | anti-p72 | pool DPI |
| --- | --- | --- | --- | --- | --- | --- | --- | --- |
| a1 | IPC1 | Est15/WB-Valga6 | moderate virulent | acute | innoculated | 7 | + | 7 |
| a2 | IPC2 | Est15/WB-Valga6 | moderate virulent | sub-acute | innoculated | 0 | - | 0 |
| a3 |  |  |  |  |  | 7 | - | 7 |
| a4 |  |  |  |  |  | 10 | + | 10 |
| a5 |  |  |  |  |  | 14 | + | 13-16 |
| a6 |  |  |  |  |  | 17 | + | 17-20 |
| a7 |  |  |  |  |  | 24 | + | 21-30 |
| a8 | CPC3 | Est15/WB-Valga6 | moderate virulent | chronic | in contact | 0 | - | 0 |
| a9 |  |  |  |  |  | 17 | + | 17-20 |
| a10 |  |  |  |  |  | 21 | + | 21-30 |
| a11 |  |  |  |  |  | 28 | + | 21-30 |
| a12 |  |  |  |  |  | 35 | + | 35-72 |
| a13 | CPC6 | Est15/WB-Valga6 | moderate virulent | sub-acute | in contact | 0 | - | 0 |
| a14 |  |  |  |  |  | 3 | - | 3 |
| a15 |  |  |  |  |  | 7 | - | 7 |
| a16 |  |  |  |  |  | 10 | - | 10 |
| a17 |  |  |  |  |  | 14 | - | 13-16 |
| a18 |  |  |  |  |  | 17 | - | 17-20 |
| a19 |  |  |  |  |  | 21 | + | 21-30 |
| a20 | IPC7 | Est15/WB-Tartu14 | moderate virulent | acute | innoculated | 0 | - | 0 |
| a21 |  |  |  |  |  | 3 | - | 3 |
| a22 |  |  |  |  |  | 7 | + | 7 |
| a23 |  |  |  |  |  | 10 | + | 10 |
| a24 | E2 | ES16/WB-Viru8 | moderate virulent | sub-acute | in contact | 0 | - | 0 |
| a25 |  |  |  |  |  | 3 | - | 3 |
| a26 |  |  |  |  |  | 7 | - | 7 |
| a27 |  |  |  |  |  | 10 | - | 10 |
| a28 |  |  |  |  |  | 13 | - | 13-16 |
| a29 |  |  |  |  |  | 16 | + | 13-16 |
| a30 |  |  |  |  |  | 20 | + | 17-20 |
| a31 |  |  |  |  |  | 27 | + | 21-30 |
| a32 |  |  |  |  |  | 30 | + | 21-30 |
| a33 |  |  |  |  |  | 37 | + | 35-72 |
| a34 |  |  |  |  |  | 44 | + | 35-72 |
| a35 |  |  |  |  |  | 52 | + | 35-72 |
| a36 |  |  |  |  |  | 58 | + | 35-72 |
| a37 |  |  |  |  |  | 65 | + | 35-72 |
| a38 |  |  |  |  |  | 72 | + | 35-72 |
| a39 | PW14 | LV17/WB/Rie1 | attenuated | chronic | in contact | 0 | - | 0 |
| a40 |  |  |  |  |  | 3 | - | 3 |
| a41 |  |  |  |  |  | 7 | - | 7 |
| a42 |  |  |  |  |  | 10 | - | 10 |
| a43 |  |  |  |  |  | 14 | + | 13-16 |
| a44 |  |  |  |  |  | 17 | + | 17-20 |
| a45 |  |  |  |  |  | 22 | + | 21-30 |
| a46 |  |  |  |  |  | 29 | + | 21-30 |
| a47 |  |  |  |  |  | 35 | + | 35-72 |
| a48 |  |  |  |  |  | 42 | + | 35-72 |
| a49 | PW17 | LV17/WB/Rie1 | attenuated | chronic | innoculated | 0 | - | 0 |
| a50 |  |  |  |  |  | 3 | - | 3 |
| a51 |  |  |  |  |  | 22 | + | 21-30 |
| a52 |  |  |  |  |  | 29 | + | 21-30 |
| a53 |  |  |  |  |  | 35 | + | 35-72 |
| a54 |  |  |  |  |  | 42 | + | 35-72 |
| a55 |  |  |  |  |  | 45 | + | 35-72 |
| a56 | IM6 | LV17/WB/Rie1 | attenuated | chronic | innoculated | 0 | - | 0 |
| a57 |  |  |  |  |  | 3 | - | 3 |
| a58 |  |  |  |  |  | 7 | - | 7 |
| a59 |  |  |  |  |  | 10 | + | 10 |
| a60 |  |  |  |  |  | 14 | + | 13-16 |
| a61 |  |  |  |  |  | 17 | + | 17-20 |
| a62 |  |  |  |  |  | 18 | + | 17-20 |
| a63 | IM7 | LV17/WB/Rie1 | attenuated | chronic | innoculated | 0 | - | 0 |
| a64 |  |  |  |  |  | 3 | - | 3 |
| a65 |  |  |  |  |  | 7 | - | 7 |
| a66 |  |  |  |  |  | 10 | + | 10 |
| a67 |  |  |  |  |  | 14 | + | 13-16 |
| a68 |  |  |  |  |  | 17 | - | 17-20 |
| a69 | IM8 | LV17/WB/Rie1 | attenuated | chronic | innoculated | 0 | - | 0 |
| a70 |  |  |  |  |  | 3 | - | 3 |
| a71 |  |  |  |  |  | 7 | - | 7 |
| a72 |  |  |  |  |  | 10 | + | 10 |
| a73 |  |  |  |  |  | 14 | + | 13-16 |
| a74 |  |  |  |  |  | 17 | + | 17-20 |
| a75 |  |  |  |  |  | 21 | + | 21-30 |
| a76 | IM9 | LV17/WB/Rie1 | attenuated | chronic | innoculated | 0 | - | 0 |
| a77 |  |  |  |  |  | 3 | - | 3 |
| a78 |  |  |  |  |  | 7 | - | 7 |
| a79 |  |  |  |  |  | 10 | + | 10 |
| a80 |  |  |  |  |  | 14 | + | 13-16 |
| a81 |  |  |  |  |  | 17 | + | 17-20 |
| a82 |  |  |  |  |  | 18 | + | 17-20 |
| b1 | M1 | Lv17/WB/Rie1 ΔEP153R | attenuated | chronic | innoculated | 19 | + | n.i. |
| b2 | M5 | Lv17/WB/Rie1 ΔEP153R | attenuated | chronic | innoculated | 19 | + | n.i. |
| b3 | M7 | Lv17/WB/Rie1 ΔUK | moderate virulent | acute | innoculated | 14 | + | n.i. |
| b4 | M8 | Lv17/WB/Rie1 ΔUK | moderate virulent | acute | innoculated | 13 | + | n.i. |
| b5 | M9 | Lv17/WB/Rie1 ΔUK | moderate virulent | acute | innoculated | 14 | + | n.i. |
| b6 | M10 | Lv17/WB/Rie1 ΔUK | moderate virulent | acute | innoculated | 16 | + | n.i. |
| b7 | M11 | Lv17/WB/Rie1 ΔUK | moderate virulent | acute | innoculated | 13 | + | n.i. |
| b8 | M12 | Lv17/WB/Rie1 ΔUK | moderate virulent | chronic | innoculated | 16 | + | n.i. |
| b9 | M14 | LV17/WB/Rie1 | attenuated | chronic | innoculated | 30 | + | n.i. |
| b10 | C7 | Lv17/WB/Rie1 | attenuated | chronic | innoculated | 14 | + | n.i. |
| b11 | ID19 | Lv17/WB/Rie1 | attenuated | chronic | innoculated | 21 | + | n.i. |
| b12 | ON23 | not infected | | | | 0 | - | n.i. |
